# Comparative transcriptomics reveals PrrAB-mediated control of metabolic, respiration, energy-generating, and dormancy pathways in *Mycobacterium smegmatis*

**DOI:** 10.1186/s12864-019-6105-3

**Published:** 2019-12-07

**Authors:** Jason D. Maarsingh, Shanshan Yang, Jin G. Park, Shelley E. Haydel

**Affiliations:** 1 School of Life Sciences, Arizona State University, Tempe, AZ USA; 2 Department of Obstetrics and Gynecology, College of Medicine-Phoenix, University of Arizona, Phoenix, AZ USA; 3 Bioinformatics Core, Knowledge Enterprise Development, Arizona State University, Tempe, AZ USA; 4 The Biodesign Institute Virginia G. Piper Center for Personalized Diagnostics, Arizona State University, Tempe, AZ USA; 5 The Biodesign Institute Center for Immunotherapy, Vaccines and Virotherapy, Arizona State University, Tempe, AZ USA

**Keywords:** *Mycobacterium smegmatis*, *Mycobacterium tuberculosis*, *prrAB*, Two-component system, RNA-seq, Transcriptomics, Hypoxia, Respiration, Oxidative phosphorylation, ATP

## Abstract

**Background:**

*Mycobacterium smegmatis* is a saprophytic bacterium frequently used as a genetic surrogate to study pathogenic *Mycobacterium tuberculosis*. The PrrAB two-component genetic regulatory system is essential in *M. tuberculosis* and represents an attractive therapeutic target. In this study, transcriptomic analysis (RNA-seq) of an *M. smegmatis* Δ*prrAB* mutant was used to define the PrrAB regulon and provide insights into the essential nature of PrrAB in *M. tuberculosis*.

**Results:**

RNA-seq differential expression analysis of *M. smegmatis* wild-type (WT), Δ*prrAB* mutant, and complementation strains revealed that during in vitro exponential growth, PrrAB regulates 167 genes (*q* < 0.05), 57% of which are induced in the WT background. Gene ontology and cluster of orthologous groups analyses showed that PrrAB regulates genes participating in ion homeostasis, redox balance, metabolism, and energy production. PrrAB induced transcription of *dosR* (*devR*), a response regulator gene that promotes latent infection in *M. tuberculosis* and 21 of the 25 *M. smegmatis* DosRS regulon homologues. Compared to the WT and complementation strains, the Δ*prrAB* mutant exhibited an exaggerated delayed growth phenotype upon exposure to potassium cyanide and respiratory inhibition. Gene expression profiling correlated with these growth deficiency results, revealing that PrrAB induces transcription of the high-affinity cytochrome *bd* oxidase genes under both aerobic and hypoxic conditions. ATP synthesis was ~ 64% lower in the Δ*prrAB* mutant relative to the WT strain, further demonstrating that PrrAB regulates energy production.

**Conclusions:**

The *M. smegmatis* PrrAB two-component system regulates respiratory and oxidative phosphorylation pathways, potentially to provide tolerance against the dynamic environmental conditions experienced in its natural ecological niche. PrrAB positively regulates ATP levels during exponential growth, presumably through transcriptional activation of both terminal respiratory branches (cytochrome c *bc*_1_-*aa*_3_ and cytochrome *bd* oxidases), despite transcriptional repression of ATP synthase genes. Additionally, PrrAB positively regulates expression of the dormancy-associated *dosR* response regulator genes in an oxygen-independent manner, which may serve to fine-tune sensory perception of environmental stimuli associated with metabolic repression.

## Background

Two-component systems (TCSs) participate in signal transduction pathways and are ubiquitously found in bacteria, archaea, some lower eukaryotes and plants [1–4]. TCSs recognize specific environmental stimuli [5] and integrate an adaptive response, frequently by modulating transcription [6]. A prototypical TCS consists of a membrane-bound histidine kinase sensor and a cytoplasmic DNA-binding response regulator. In pathogenic bacteria, TCSs act as virulence factors that regulate diverse survival mechanisms, such as antibiotic resistance [7], phosphate limitation [8], low oxygen tension [9], and evasion of immune responses [10]. Though mammalian proteins bearing histidine kinase sequence motifs and activity [11] have been identified, response regulators appear absent in humans, opening the possibility for development of inhibitors targeting virulence-related or essential bacterial TCSs as novel therapeutic approaches.

*Mycobacterium tuberculosis*, the causative agent of tuberculosis, is an ancient disease of mankind and the leading cause of death from an infectious agent [12]. The *M. tuberculosis* genome harbors 11 paired TCSs, two orphaned histidine kinases, and six orphaned response regulators [13]. Of these TCSs, only MtrAB [14] and PrrAB [15] are essential for *M. tuberculosis* viability. The *prrA* response regulator and *prrB* histidine kinase genes are conserved across all fully-sequenced mycobacterial genomes, suggesting an evolutionary selective pressure to retain these TCS genes. *M. tuberculosis prrAB* is upregulated during the early stages of human macrophage infection [13] and under in vitro nitrogen limitation [15]. During infection in murine macrophages, *prrAB* is required for early replication and adaptation to the intracellular environment [16]. Capitalizing on findings that diarylthiazole compounds inhibit *M. tuberculosis* growth via the PrrAB TCS, Bellale et al. [17] exposed *M. tuberculosis* cultures to diarylthiazole and found that PrrAB modulates transcription of genes enabling metabolic adaptation to a lipid-rich environment, responsiveness to reduced oxygen tension, and production of essential ribosomal proteins and amino acid tRNA synthases.

*Mycobacterium smegmatis* strain mc^2^155 [18] is a non-pathogenic, rapid-growing, saprophytic mycobacterium that is used as a surrogate model to study *M. tuberculosis* genetics and mycobacterial TCSs. We recently demonstrated that *prrAB* is not essential in *M. smegmatis* and that PrrAB differentially regulates triacylglycerol biosynthetic genes during ammonium limitation [19]. The inability to generate an *M. tuberculosis prrAB* knockout mutant [15], the high degree of PrrA sequence identity (95%) between *M. tuberculosis* and *M. smegmatis*, and the presence of over 2000 homologous genes (51% of total genes in *M. tuberculosis* H37Rv) shared between these species prompted use of the *M. smegmatis* Δ*prrAB* mutant to better understand PrrAB transcriptional regulatory properties. A comprehensive profiling of the genes and pathways regulated by PrrAB in *M. smegmatis* would provide insights into the genetic adaptations that occur during *M. tuberculosis* infection and open new avenues for discovering novel therapeutic targets to treat tuberculosis.

In this study, we used RNA-seq-based transcriptomics analysis to obtain a global profile of the genes regulated by PrrAB in *M. smegmatis*. We compared the transcriptomic profiles of *M. smegmatis* WT, Δ*prrAB* mutant, and *prrAB* complementation strains during mid-logarithmic growth under standard laboratory conditions. Genes repressed by PrrAB were associated with broad aspects of metabolism and components of the F_1_F_0_ ATPase, while PrrAB induced genes involved in oxidoreductase activity, respiration, hypoxic response, and ion homeostasis. These data provide seminal information into the transcriptional regulatory properties of the mycobacterial PrrAB TCS and how PrrAB may be controlling molecular processes important in *M. tuberculosis* and other mycobacteria.

## Results

### Phylogenetic analyses of PrrA and PrrB in mycobacteria

**Fig. 1:**
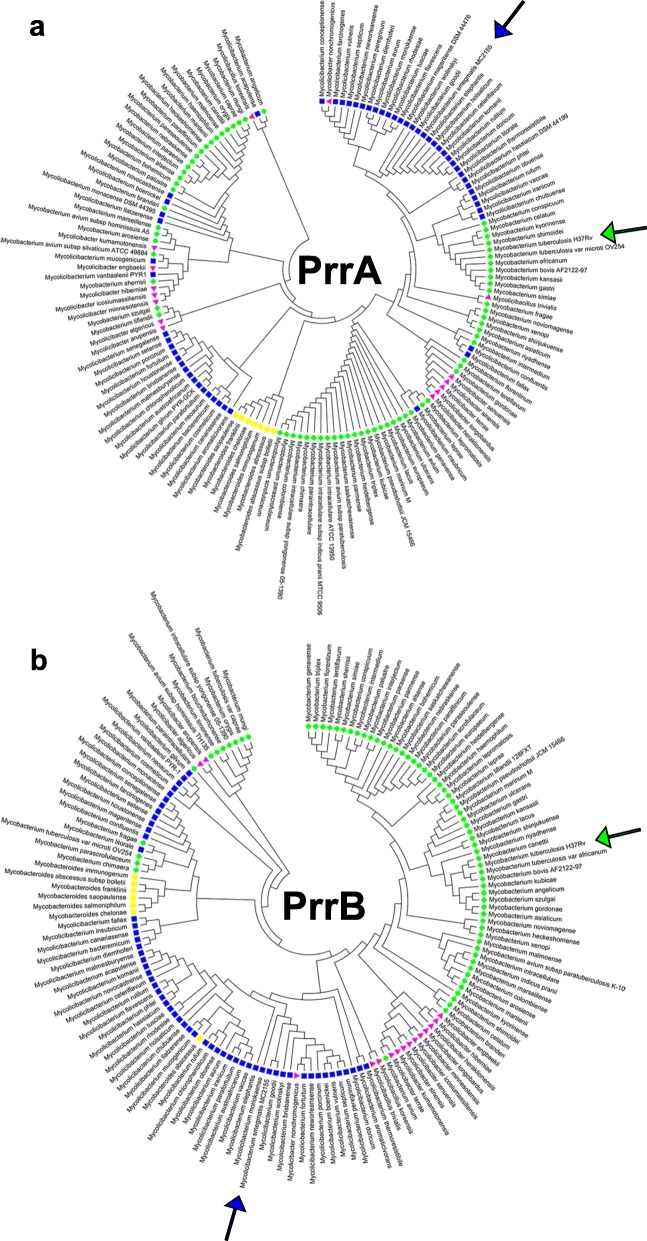
Maximum-likelihood phylogenetic analyses of mycobacterial (**a**) PrrA and (**b**) PrrB sequences based on the recent reclassification of mycobacterial species by Gupta et al. [20]. Blue squares, *Fortuitum-Vaccae* clade. Red triangles, *Trivale* clade. Green diamonds, *Tuberculosis-Simiae* clade. Yellow circles, *Abscessus-Chelonae* clade. Purple triangles, *Terrae* clade. *M. smegmatis* mc^2^155 and *M. tuberculosis* H37Rv are indicated by blue and green arrows, respectively. PrrA and PrrAB sequences were aligned using default MUSCLE algorithms [21] and phylogenetic tree was generated in MEGA 7 [22]

Since *prrAB* orthologues are present in all mycobacterial species and *prrAB* is essential for viability in *M. tuberculosis* [15], it is reasonable to believe that PrrAB fulfills important regulatory properties in mycobacteria. We therefore questioned the evolutionary relatedness or distance between PrrA and PrrB proteins in mycobacteria. The *M. tuberculosis* H37Rv and *M. smegmatis* mc^2^155 PrrA and PrrB amino acid sequences share 93 and 81% identity, respectively. Maximum-likelihood phylogenetic trees, based on PrrA (Fig. 1a) and PrrB (Fig. 1b) multiple sequence alignments, were generated. Using the Gupta et al. [20] recent reclassification of mycobacterial species, the results suggested that, with a few exceptions, PrrA and PrrB evolved with specific mycobacterial clades (Fig. 1). While subtle differences in the PrrA or PrrB sequences may represent evolutionary changes as mycobacterial species of the same clade adapted to similar environmental niches, additional experiments are needed to determine if *prrAB* is essential in other pathogenic mycobacteria.

We next questioned if the distinct phylogenetic separations between clades could be mapped to specific PrrA or PrrB amino acid residues. We separately aligned mycobacterial PrrA and PrrB sequences in JalView using the default MUSCLE algorithm [21]. Within species of the *Abscessus-Chelonae* clade, two unique PrrA signatures were found: asparagine and cysteine substitutions relative to serine 38 (S38) and serine 49 (S49), respectively, of the *M. smegmatis* PrrA sequence (See Additional file 1: Figure S1). These *Abscessus-Chelonae* clade PrrA residues were not found at similar aligned sites in other mycobacteria (See Additional file 1: Figure S1). Similarly, members of the *Abscessus-Chelonae* clade (except *Mycobacteriodes abscessus*) harbored unique amino acid substitutions in PrrB, including glutamate, valine, lysine, aspartate, lysine, and valine corresponding to threonine 42 (T42), glycine 67 (G67), valine 90 (V90), methionine 318 (M318), alanine 352 (A352), and arginine (R371), respectively, of the *M. smegmatis* PrrB sequence (See Additional file 1: Figure S2).

### Transcriptomics analysis of the *M. smegmatis* WT, Δ*prrAB* mutant, and complementation strains

We previously generated an *M. smegmatis* mc^2^155 *prrAB* deletion mutant (mc^2^155::Δ*prrAB*; FDL10) and its complementation strain (mc^2^155::Δ*prrAB*::*prrAB*; FDL15) [19]. Since the *prrAB* regulon and the environmental cue which stimulates PrrAB activity are unknown, a global transcriptomics approach was used to analyze differential gene expression in standard laboratory growth conditions. RNA-seq was used to determine transcriptional differences between the Δ*prrAB* mutant, mc^2^155, and the complementation strains during mid exponential growth, corresponding to an OD_600_ of ~ 0.6 (See Additional file 1: Figure S3), in supplemented Middlebrook 7H9 (M7H9) broth. Total RNA was isolated from three independent, biological replicates of each *M. smegmatis* strain. Based on multidimensional scaling (MDS) plot, one mc^2^155 biological replicate was deemed an outlier and excluded from subsequent analyses (details in Methods, see Additional file 1: Figure S4). Principal component analysis of the global expression patterns of the samples demonstrated that samples from the mc^2^155 and FDL15 complementation strains clustered together, apart from those of the FDL10 Δ*prrAB* strain with the majority of variance occurring along PC1 (See Additional file 1: Figure S5), indicating complementation with ectopically-expressed *prrAB* in the Δ*prrAB* background.

### Identifying the PrrAB regulon

**Fig. 2:**
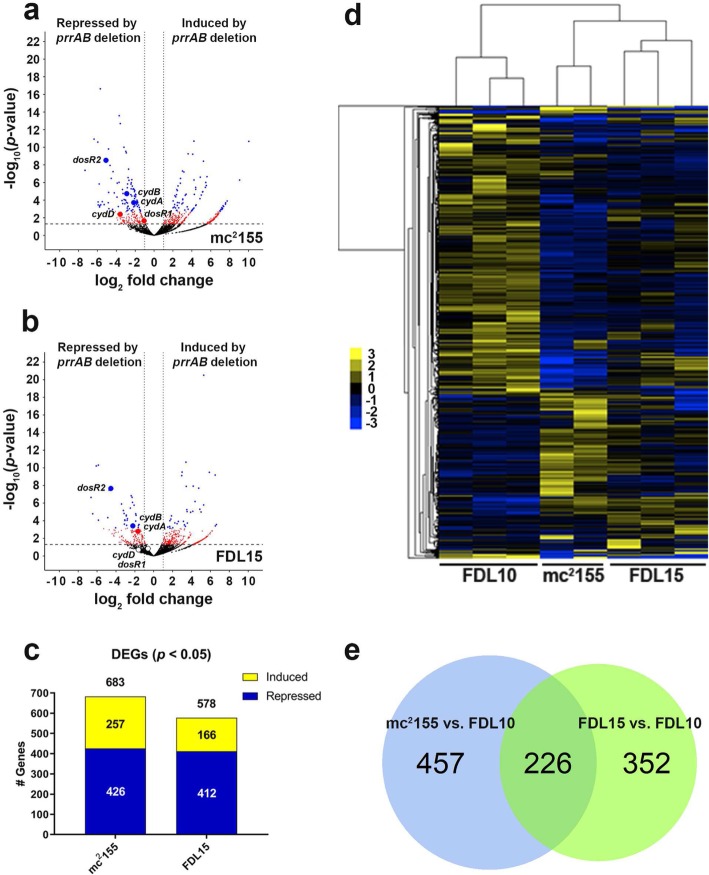
Global DEG profiles (*q* < 0.05) between the mc^2^155 vs. FDL10 and FDL15 vs. FDL10 RNA-seq comparisons. Volcano plots of (**a**) FDL10 vs. mc^2^155 and (**b**) FDL10 vs. FDL15 group comparisons with red and blue dots representing differentially-expressed genes with *p* < 0.05 and *q* < 0.05, respectively. The horizontal hatched line indicates *p* = 0.05 threshold, while the left and right vertical dotted lines indicate log_2_ fold change of − 1 and + 1, respectively. **c** Repressed (blue) and induced (yellow) DEGs (*q <* 0.05) in mc^2^155 (WT) and FDL15 (*prrAB* complementation strain) compared to the FDL10 Δ*prrAB* mutant. **d** Average hierarchical clustering (FPKM + 1) of individual RNA-seq sample replicates. **e** Venn diagrams indicating 40 overlapping DEGs (*q* < 0.05) between mc^2^155 vs. FDL10 (WT vs. Δ*prrAB* mutant) and FDL15 vs. FDL10 (*prrAB* complementation strain vs. Δ*prrAB* mutant) strain comparisons

To identify differentially-expressed genes (DEGs), pair-wise comparisons of normalized read counts between the Δ*prrAB* mutant and WT (FDL10 vs. mc^2^155) as well as the Δ*prrAB* mutant and *prrAB* complementation (FDL10 vs. FDL15) datasets were performed using EdgeR. Deletion of *prrAB* resulted in induction of 95 genes and repression of 72 genes (*q* < 0.05), representing 167 transcriptional targets (Fig. 2a) that are repressed and induced, respectively, by PrrAB in the WT background (Fig. 2c). Less conservative comparisons revealed 683 DEGs (*p* < 0.05) between the WT and Δ*prrAB* mutant strains (See Additional file 1: Figure S6a). Between the Δ*prrAB* complementation and *prrAB* mutant strains, 67 DEGs (*q* < 0.05) were identified (Fig. 2b), representing 35 repressed and 32 induced genetic targets by the complementation of PrrAB (Fig. 2c), while less conservative comparisons (*p* < 0.05) revealed 578 DEGs (See Additional file 1: Figure S6a). Overall, pair-wise DEG analyses revealed that during mid-logarithmic *M. smegmatis* growth, PrrAB regulates transcription through a relatively balanced combination of gene induction and repression. In addition, comparison between the two DEG sets (i.e., for mc^2^155 vs. FDL10 and FDL15 vs. FDL10) datasets revealed 40 (Fig. 2e) and 226 (See Additional file 1: Figure S6b) overlapping DEGs at the significance levels of *q* < 0.05 and *p* < 0.05, respectively. Hierarchical clustering with the overlapping DEGs further illustrated that gene expression changes induced by the *prrAB* deletion were partially recovered by *prrAB* complementation (Fig. 2d). We randomly selected six DEGs for qRT-PCR analyses and verified the RNA-seq results for five genes in both the FDL10 vs. mc^2^155 and FDL10 vs. FDL15 comparisons (See Additional file 1: Figure S7). [See Additional file 2 for a complete list of DEGs between all pair-wise comparisons.]

### Gene ontology and clustering analyses

**Fig. 3:**
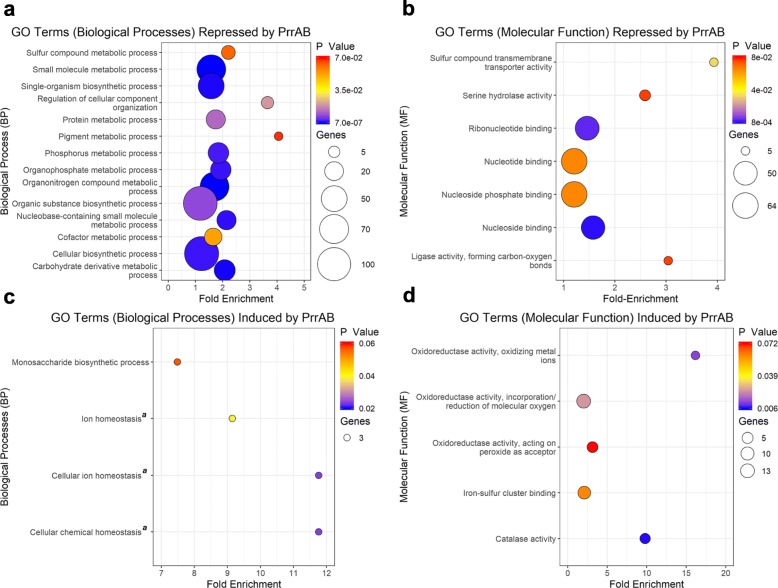
GO term enrichment associated with DEGs (*p* < 0.05) that are (**a, b**) repressed (**c, d**) or induced by PrrAB in the WT background. GO terms categorized by (**a, c**) biological processes (BP) or (**b, d**) molecular function (MF). ^*a*^ GO terms share a common set of genes: *MSMEG 3564, MSMEG 6422,* and *MSMEG 6467*

To infer function of the genes regulated by PrrAB, enrichment of gene ontology (GO) terms (biological processes and molecular functions) in the DEGs of the mc^2^155 vs. FDL10 comparison was assessed by the DAVID functional annotation tool (See Additional file 3 for a complete list of functional annotations returned from the DAVID results). The two sets of DEGs from the mc^2^155 vs. FDL10 comparison (See Additional file 1: Figure S6) were examined. In general, genes repressed by PrrAB were associated with numerous metabolic processes (Fig. 3a) and nucleotide binding (Fig. 3b), while PrrAB-induced genes were associated with ion or chemical homeostasis (Fig. 3c) and oxidoreductase, catalase, and iron-sulfur cluster binding activities (Fig. 3d). Similar GO enrichment terms in the two group comparisons (mc^2^155 vs. FDL10 and FDL15 vs. FDL10) suggested evidence of genetic complementation (Fig. 3; Additional file 1: Figure S8). GO term enrichment was also found for metabolism, nucleotide binding, oxidoreductase, and catalase activity, based on conservative (*q* < 0.05) DEG comparisons (See Additional file 1: Figures S9 and S10). The GO enrichment analyses suggested that during *M. smegmatis* exponential growth in M7H9 medium, PrrAB negatively regulates genes associated with diverse components of metabolic and biosynthetic processes and positively regulates expression of genes participating in respiration (*qcrA, cydA,* and *cydB*), ion transport (via the F_1_F_0_ ATPase), redox mechanisms, and recognition of environmental signals (*dosR2*) (Fig. 3; Additional file 1: Figures S8, S9, and S10).

**Fig. 4:**
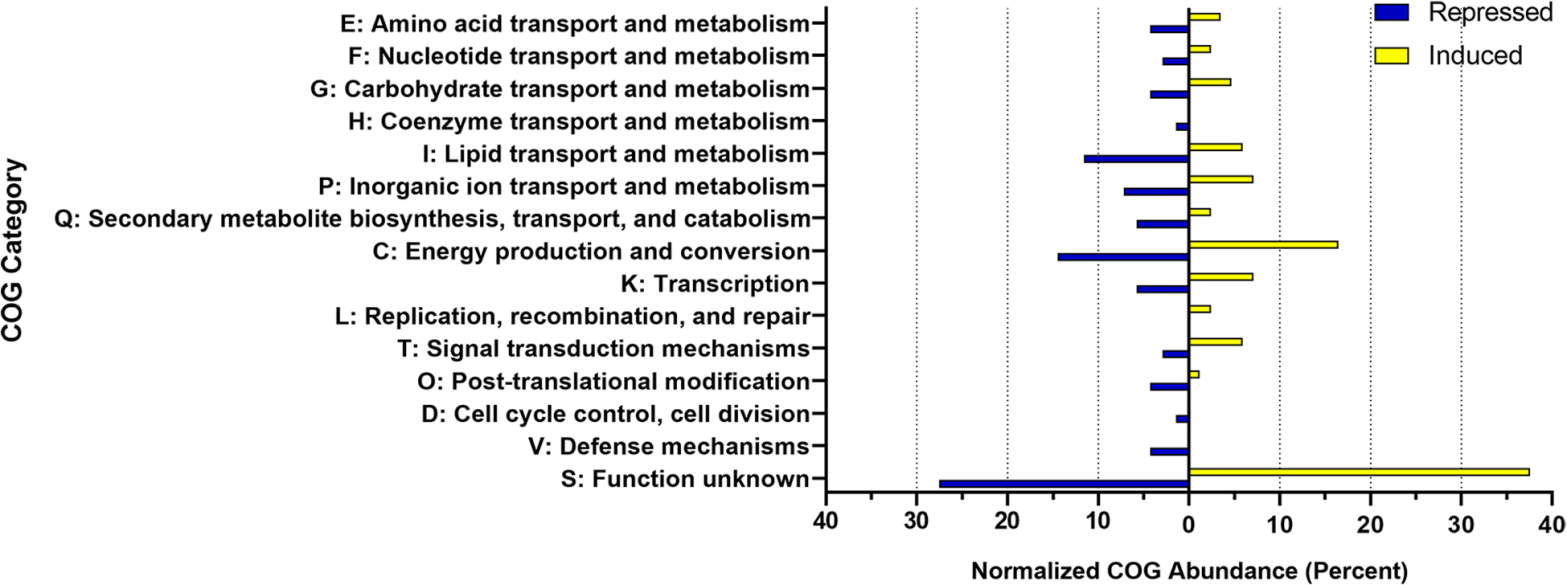
COG analysis of DEGs (*q* < 0.05) induced (yellow) or repressed (blue) by PrrAB in the WT background. COGs from each category were normalized to represent the percent abundance of each category to all COGs returned in the induced or repressed analyses, respectively

Classification of genes (*q* < 0.05) based on clusters of orthologous groups (COGs) analyses were then performed using the online eggNOG mapper program. Of all COG categories in each gene list, 32% (*n* = 22) and 24% (*n* = 20) of genes repressed or induced by PrrAB, respectively, participate in diverse aspects of metabolism (Fig. 4), thus corroborating the GO results. Of the COG categories induced by PrrAB, 17% (*n* = 14) were associated with energy production and conversion (COG Category C). The relatively even proportions of COG categories associated with PrrAB-induced and repressed genes (Fig. 4) suggest that this TCS, as both transcriptional activator and repressor, fine-tunes diverse cellular functions to maximize and/or optimize growth potential during exponential replication.

### PrrAB regulates *dosR* expression in *M. smegmatis*

**Fig. 5:**
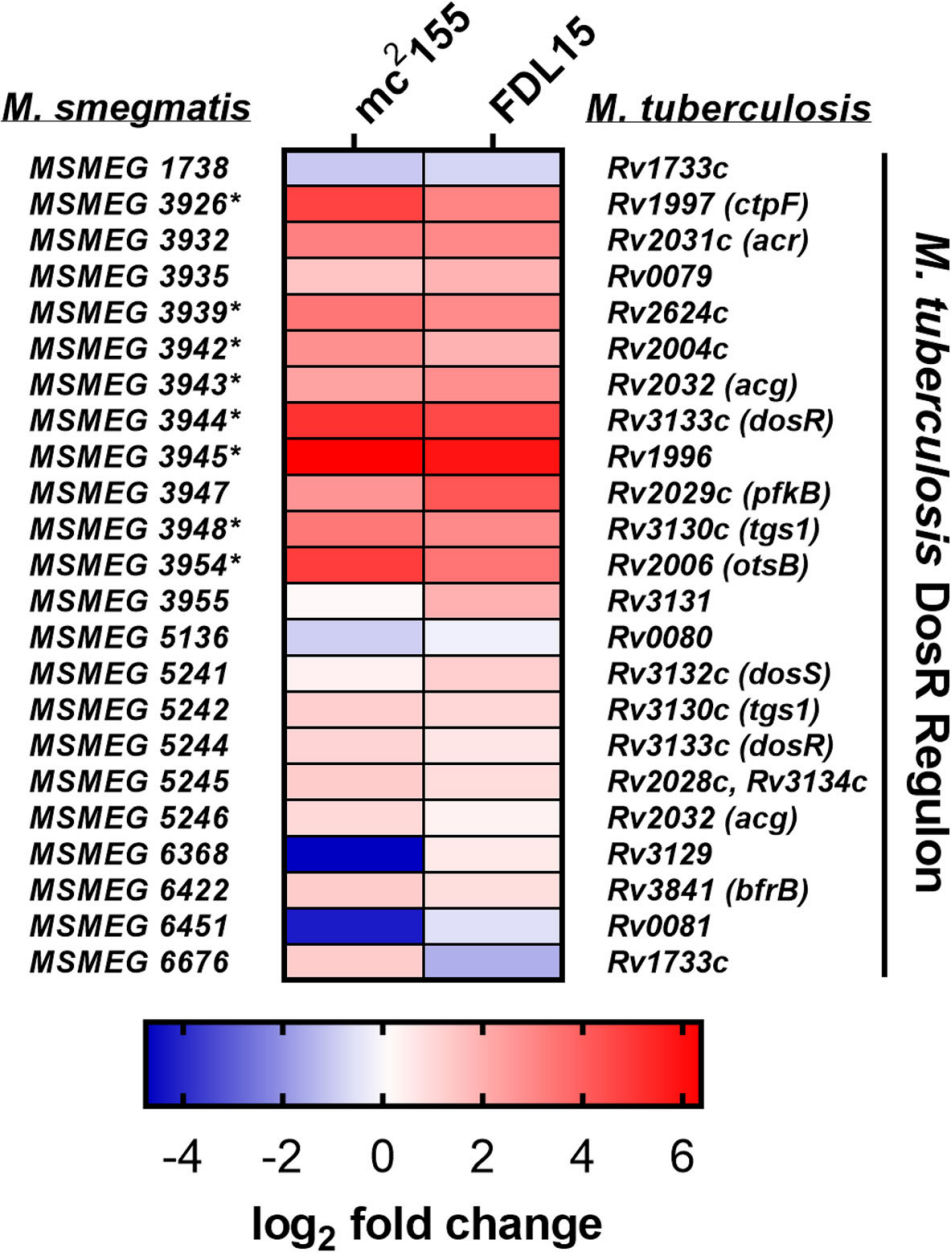
*M. smegmatis* PrrAB regulates dormancy-associated genes of the DosR regulon. Heatmap of *M. smegmatis* RNA-seq DEGs associated with *M. tuberculosis dosR* regulon homologues. Color bar indicates log_2_ fold change values corresponding to mc^2^155 vs. FDL10 (left tiles) and FDL15 vs. FDL10 (right tiles) DEGs. *M. smegmatis* genes differentially regulated (*q* < 0.05) are denoted by asterisks

Differential expression analysis revealed significant repression of *MSMEG 5244* and *MSMEG 3944*, two orthologues of the *dosR* (*devR*) response regulator gene, in the Δ*prrAB* mutant strain (Fig. 2a). In *M. tuberculosis*, the hypoxia-responsive DosRS (DevRS) TCS (along with the DosT histidine kinase) induces transcription of ~ 50 genes that promote dormancy and chronic infection [23]. Here, we designate *MSMEG 5244* as *dosR1* (due to its genomic proximity to *dosS*) and *MSMEG 3944* as *dosR2*. Among the 25 *M. smegmatis* homologues of the *M. tuberculosis* DosRS regulon genes, 7 genes were differentially expressed (+ 2-fold changes, *q* < 0.05) in pair-wise comparisons among the three strains (Fig. 5 and Additional file 4). Importantly, each of these *M. smegmatis* DosRS regulon homologues were induced by PrrAB in the WT and complementation backgrounds, corroborating the activity of the DosR as a positive transcriptional regulator [23].

### PrrAB contributes to *M. smegmatis* adaptation to hypoxia

The cytochrome *bd* oxidase respiratory system is a high-affinity terminal oxidase that is important for *M. smegmatis* survival under microaerophilic conditions [24]. Because the *cydA, cydB,* and *cydD* genes were repressed in the Δ*prrAB* mutant during aerobic growth (Fig. 2a; Additional file 2), we questioned if the Δ*prrAB* mutant was more sensitive to hypoxia than the WT strain. Compared to WT and the *prrAB* complementation strains, the Δ*prrAB* mutant exhibited reduced viability (See Additional file 1: Figure S11a) and produced smaller colonies (See Additional file 1: Figure S11b) after 24 h hypoxia exposure. In contrast, cell viability and colony sizes were similar for all strains cultured under aerobic growth conditions (See Additional file 1: Figure S11).

**Fig. 6:**
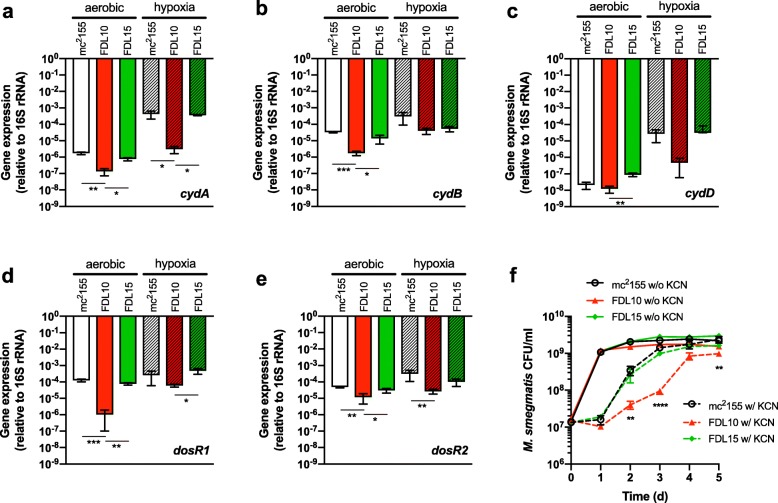
PrrAB regulates cytochrome *bd* and *dosR* expression and is protective during hypoxia and cyanide-mediated respiratory inhibition. qRT-PCR of (**a**) *cydA* (*MSMEG 3233*)*,* (**b**) *cydB* (*MSMEG 3232*)*,* (**c**) *cydD* (*MSMEG 3231*), (**d**) *dosR1* (*MSMEG 5244*), and (**e**) *dosR2* (*MSMEG 3944*) RNA isolated from *M. smegmatis* strains cultured under aerobic (solid bars) or hypoxic (hatched bars) conditions for 24 h. Relative gene expression was calculated using the 2^-ΔCt^ method and normalized to 16S rRNA for each strain and growth condition. qRT-PCR measurements for each gene and each condition (aerobic or hypoxic growth) was assessed in triplicate. *, *p* < 0.05; **, *p* < 0.01; ***, *p* < 0.001; one-way ANOVA, Dunnett’s multiple comparisons. (**f**) *M. smegmatis* growth in the presence (dashed lines) or absence (solid lines) of 1 mM cyanide (KCN). **, *p* < 0.01; ****, *p* < 0.0001; unpaired Student’s t tests. Values represent the mean ± SEM of data collected from three independent cultures

Next, we questioned if differential expression of *cydA, cydB,* and *cydD* correlated with growth deficiencies in the Δ*prrAB* mutant during hypoxia. We compared transcriptional profiles of *cydA, cydB,* and *cydD* by qRT-PCR from each strain incubated in M7H9 broth under hypoxic and aerobic conditions for 24 h. After 24 h hypoxia, *cydA* and *dosR2* expression was significantly decreased approximately 100-fold and 10-fold, respectively, in the Δ*prrAB* mutant relative to the WT strain (Fig. 6a, e). Expression levels of *cydA* and *cydB* were significantly reduced in the Δ*prrAB* mutant relative to the WT strain during aerobic growth (Fig. 6a, b). Furthermore, both *dosR1* and *dosR2* were significantly downregulated in the Δ*prrAB* mutant under aerobic conditions (Fig. 6d, e), further verifying the RNA-seq data (Additional file 2) and PrrAB-mediated regulation in both oxygen-rich and oxygen-poor environmental conditions.

### The Δ*prrAB* mutant is hypersensitive to cyanide exposure

Cyanide is a potent inhibitor of the *aa*_3_ cytochrome c oxidase in bacteria. Conversely, cytochrome *bd* oxidases in *Escherichia coli* [25], *Pseudomonas aeruginosa* [26]*,* some staphylococci [27], and *M. smegmatis* [24] are relatively insensitive to cyanide inhibition*.* In the absence of alternative electron acceptors (e.g., nitrate and fumarate), aerobic respiratory capacity after cyanide-mediated inhibition of the *M. smegmatis aa*_3_ terminal oxidase would be provided by the cytochrome *bd* terminal oxidase (CydAB). Because *cydA, cydB,* and *cydD* were significantly repressed in the Δ*prrAB* mutant (Fig. 2a), as were most subunits of the cytochrome c *bc*_*1*_ – *aa*_3_ respiratory oxidase complex (See Additional file 2), we hypothesized that the Δ*prrAB* mutant would be hypersensitive to cyanide relative to the WT and complementation strains. Cyanide inhibited all three strains during the first 24 h (Fig. 6f). While the WT and complementation strains entered exponential growth after 24 h of cyanide exposure, the Δ*prrAB* mutant exhibited significantly delayed and slowed growth between 48 and 72 h (Fig. 6f). These data demonstrated that the Δ*prrAB* mutant strain had defects in alternative cytochrome *bd* terminal oxidase pathways, further supporting that genes controlling cytochrome c *bc*_1_ and *aa*_3_ respiratory oxidases are induced by PrrAB.

### PrrAB positively regulates ATP levels

**Fig. 7:**
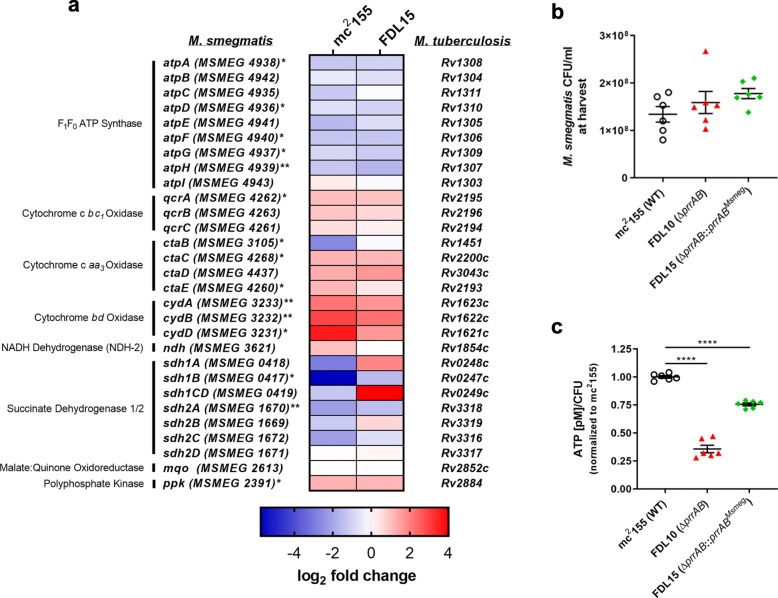
PrrAB regulates oxidative phosphorylation genes and ATP levels in *M. smegmatis*. **a** Heatmap of genes participating in oxidative phosphorylation. Color bar indicates log_2_ fold change of gene expression between mc^2^155 vs. FDL10 (left column) and FDL15 vs. FDL10 (right column). *M. smegmatis* genes significantly regulated are denoted by asterisks (*, *p* < 0.05; **, *q* < 0.05) in at least one group comparison. **b** *M. smegmatis* viability (CFU/ml) at harvest and (**c**) corresponding ATP levels (pM/CFU) normalized to mc^2^155 were measured from exponentially-growing (OD_600_ ~ 0.6) aerobic cultures in M7H9 broth. ****, *p* < 0.0001; one-way ANOVA, Dunnett’s multiple comparisons

KEGG pathway analysis of DEGs (*p* < 0.05) induced by PrrAB revealed oxidative phosphorylation as a significantly enriched metabolic pathway (Additional file 3; enrichment = 3.78; *p* = 0.017). Further examination of the RNA-seq data generally revealed that genes of the terminal respiratory complexes (cytochrome c *bc*_1_*-aa*_3_ and cytochrome *bd* oxidases) were induced by PrrAB, whereas F_1_F_0_ ATP synthase genes were repressed by PrrAB (Fig. 7a). Therefore, we hypothesized that ATP levels would be greater in the Δ*prrAB* mutant relative to the WT and complementation strains despite the apparent downregulation of terminal respiratory complex genes (except *ctaB*) in the Δ*prrAB* mutant (Fig. 7a). While viability was similar between strains at the time of sampling (Fig. 7b), ATP levels ([ATP] pM/CFU) were 36 and 76% in the Δ*prrAB* mutant and complementation strains, respectively, relative to the WT strain (Fig. 7c). Ruling out experimental artifacts, we confirmed sufficient cell lysis with the BacTiter-Glo reagent (See Methods) and that normalized extracellular ATP in cell-free supernatants were similar to intracellular ATP levels (See Additional file 1: Figure S12). These data suggested that PrrAB positively regulates ATP levels during aerobic logarithmic growth, although *prrAB* complementation did not fully restore ATP to WT levels (Fig. 7c). Additionally, ATP levels correlated with PrrAB induction of respiratory complex genes rather than PrrAB-mediated repression than F_1_F_0_ ATP synthase genes (Fig. 7a). To verify the RNA-seq data which indicates PrrAB repression of nearly all F_1_F_0_ ATP synthase genes (Fig. 7a), we directly measured transcription of three genes in the *atp* operon: *atpC* (*MSMEG 4935*)*, atpH* (*MSMEG 4939*)*,* and *atpI* (*MSMEG 4943*).

The qRT-PCR results revealed that PrrAB represses *atpC*, *atpH*, and *atpI* in the WT and *prrAB* complementation strains (See Additional file 1: Figure S13).

## Discussion

TCSs provide transcriptional flexibility and adaptive responses to specific environmental stimuli in bacteria [28]. The mycobacterial PrrAB TCS is conserved across most, if not all, mycobacterial lineages and is essential for viability in *M. tuberculosis* [15], thus representing an attractive therapeutic target [17]. Here, we use an *M. smegmatis* Δ*prrAB* mutant [19] as a surrogate to provide insights into the essential nature and regulatory properties associated with the PrrAB TCS in *M. tuberculosis*. Our rationale for this approach is founded on the high degree of identity between the *M. smegmatis* and *M. tuberculosis* PrrA and PrrB sequences, including 100% identity in the predicted DNA-binding recognition helix of PrrA (See Additional file 1: Figure S14) [29].

Using BLAST queries of *M. smegmatis* PrrA and PrrB against 150 recently reclassified mycobacterial species, as proposed by Gupta et al. [20], all fully-sequenced mycobacterial genomes harbored *prrA* and *prrB* homologues, implying strong evolutionary conservation for the PrrAB TCS. Likely due to the incomplete genomic sequences [20], *prrA* was not found in *Mycobacterium timonense* and *Mycobacterium bouchedurhonense* genomes, while a *prrB* homolog was not identified in *Mycobacterium avium subsp. silvaticum*. Phylogenetic analyses showed that PrrA and PrrB sequences grouped closely, but not perfectly, within members of specific mycobacterial clades (Fig. 1), and members of the *Abscessus-Chelonae* clade harbored unique PrrA and PrrB amino acid substitutions (See Additional file 1: Figures S1 and S2). While it is unclear if these residues impact PrrA or PrrB functionality in the *Abscessus-Chelonae* clade, it may be possible to develop *prrAB*-based single nucleotide polymorphism genotyping or proteomic technologies for differentiating mycobacterial infections. Multiple sequence alignments of the *M. smegmatis* and *M. tuberculosis* PrrA DNA-binding recognition helices revealed 100% sequence conservation (See Additional file 1: Figure S14), suggesting a shared set of core genes regulated by PrrA in mycobacteria. Incorporation of a global approach, such as ChIP-seq, will be valuable for identifying and characterizing the essential genes directly regulated by PrrA in *M. tuberculosis* and other mycobacterial species.

We used RNA-seq-based transcriptomics analyses to define the *M. smegmatis* PrrAB regulon during exponential growth under standard laboratory conditions. We showed that in *M. smegmatis,* PrrAB deletion led to differential expression of 167 genes (*q* < 0.05), corresponding to ~ 2% of chromosomal genes, of which 95 genes are induced and 72 are repressed in the WT background (Fig. 2). Importantly, PrrAB differentially-regulated genes were involved in aerobic and microaerophilic respiration. The cytochrome c terminal oxidase *bc*_1_ (*qcrCAB)* and *aa*_3_ (*ctaC*) genes are essential in *M. tuberculosis,* but not in *M. smegmatis*, and mutants in the latter species are attenuated during exponential phase growth [30]. If *M. tuberculosis* PrrAB also regulates genes of the cytochrome c *bc*_1_ and/or *aa*_3_ respiratory complex, it could partially explain *prrAB* essentiality.

To corroborate the key findings from comparing the Δ*prrAB* mutant and WT strains, we included the *prrAB* complementation strain in our RNA-seq analyses. Of the 683 DEGs (*p* < 0.05) that were affected by the Δ*prrAB* mutation, expression changes of 10 genes were variably reversed in the Δ*prrAB* complementation strain. Induction of the three genes (*MSMEG 5659*, *MSMEG 5660*, and *MSMEG 5661*) adjacent to *prrAB* could be related to alteration of regulatory control sequences during generation of the knockout mutation. These results were unlikely due to poor RNA quality, as RNA integrity numbers (RIN) were consistently high (Additional file 5). We previously demonstrated similar *prrA* transcription and PrrA protein levels in the WT and complementation strains during aerobic mid-logarithmic growth in M7H9 broth [19], similar to the growth conditions employed in this study. The lack of full complementation seen in our RNA-seq results is likely affected by the low number of biological replicates analyzed. Baccerella et al. [31] demonstrated that sample number impacts RNA-seq performance to a greater degree relative to read depth. Although we found only 226 overlapping DEGs (*p* < 0.05) between the mc^2^155 vs. FDL10 and FDL15 vs. FDL10 group comparisons, global DEG regulation (i.e., relative ratios of up- or down-regulated genes), was similar. In both pairwise comparisons, 32 and 36% of all DEGs were induced by PrrAB in the WT and complementation backgrounds, respectively, while 68 and 64% of all DEGs were repressed by PrrAB in the WT and complementation backgrounds, respectively. These data indicate that complementation with *prrAB* in the deletion background restored global transcriptomic profiles to WT levels. Including additional biological replicates will improve the statistical reliability for better comparison of wild-type and complementation strains which, to the best of our knowledge, has not been previously reported in a transcriptomic study.

We found 40 DEGs (*q* < 0.05) that overlapped between the WT vs. Δ*prrAB* mutant and complementation vs. Δ*prrAB* mutant group comparisons (Fig. 2e). In this data set, enrichment analysis for the GO term “response to stimulus” contains genes of the DosR regulon (Additional file 6). A less-conservative approach using 226 overlapping DEGs (*p* < 0.05) revealed enrichment in GO terms related to respiratory pathways and ATP synthesis (Additional file 6), therefore corroborating our phenotypic and biochemical data (Figs. 6 and 7). It is interesting to postulate that these DEGs may accurately represent the PrrAB regulon in *M. smegmatis* under the conditions tested, as they are significantly represented in both WT and complementation strain group comparisons. Future studies are warranted to explore the utility of incorporating sequencing data from both WT and complementation strains to improve the reliability of transcriptomics experiments.

*M. tuberculosis* acclimates to an intramacrophage environment and the developing granuloma by counteracting the detrimental effects of hypoxia [32], nutrient starvation [33], acid stress [34], and defense against reactive oxygen and nitrogen species [35]. Adaptive measures to these environmental insults include activation of the dormancy regulon and upregulation of the high-affinity cytochrome *bd* respiratory oxidase [35], induction of the glyoxylate shunt and gluconeogenesis pathways [36], asparagine assimilation [37], and nitrate respiration [38]. As a saprophytic bacterium, *M. smegmatis* could encounter similar environmental stresses as *M. tuberculosis*, despite their drastically different natural environmental niches. Conserving the gene regulatory circuit of the PrrAB TCS for adaptive responses would thus be evolutionarily advantageous.

The hypoxia-responsive DosRS TCS controls the dormancy regulon in both *M. tuberculosis* [23] and *M. smegmatis* [39–41]. The *M. smegmatis* DosRS TCS regulates dormancy phenotypes similar to *M. tuberculosis*, including upregulation of the *dosRS* TCS [39], gradual adaptation to oxygen depletion [42], and upregulation of alanine dehydrogenase [43]. DosR is required for optimal viability in *M. smegmatis* after the onset of hypoxia [41]. Our RNA-seq and qRT-PCR data revealed that PrrAB induces both *M. smegmatis dosR* homologues (*dosR1* and *dosR2*) during aerobic and hypoxic growth (Additional file 2, Fig. 2a, Fig. 6d, and Fig. 6e). Additionally, the RNA-seq data revealed that PrrAB induces genes associated with the *M. tuberculosis* DosR regulon [23, 44] (Fig. 5). Thus, it is possible that PrrAB also positively regulates *dosR* expression in *M. tuberculosis*, which would provide additional mechanisms of *dosR* control as previously demonstrated with PknB [45], PknH [46], NarL [47], and PhoP [48].

The *M. tuberculosis* respiration and oxidative phosphorylation pathways have increasingly gained attention as promising anti-tuberculosis therapeutic targets. Bedaquiline (TMC207), a recent FDA-approved mycobacterial F_1_F_0_ ATP synthase inhibitor, is active against drug-sensitive and drug-resistant *M. tuberculosis* strains [49, 50], as is Q203 (telacebec), a cytochrome c *bc*_1_ inhibitor, which has advanced to Phase 2 clinical trials [51]. Accumulating evidence suggests that the alternative terminal cytochrome *bd* oxidase system, encoded by the *cydABDC* genes in *M. tuberculosis*, is important during chronic infection and may represent a novel drug target. *M. tuberculosis cydA* mutants are hypersensitive to the bactericidal activity of bedaquiline [52], suggesting that combined therapeutic regimens simultaneously targeting the F_1_F_0_ ATP synthase and cytochrome *bd* oxidase represent promising anti-tuberculosis treatment strategies. Analysis of the DEGs (*p* < 0.05) induced by PrrAB (Additional file 3) revealed significant enrichment of the oxidative phosphorylation KEGG pathway, including genes encoding the cytochrome c *bc*_1_ (*qcrA*), cytochrome c *aa*_3_ (*ctaC, ctaE*), and cytochrome *bd* (*cydB*, *cydD*) terminal respiratory branches. We showed that the Δ*prrAB* mutant was more sensitive to hypoxic stress and cyanide inhibition relative to the WT and complementation strains (See Additional file 1: Figure S11 and Fig. 6), thus corroborating the transcriptomics results. Although 24 h hypoxia only caused a modest reduction in the Δ*prrAB* mutant after 24 h hypoxia exposure, relative to the WT and complementation strains, the Δ*prrAB* mutant small colony phenotype indicated a growth defect under these conditions (See Additional file 1: Figure S11). Additionally, qRT-PCR results demonstrated significantly lower expression of *cydA* and *dosR2* in the Δ*prrAB* mutant relative to WT during hypoxic growth, further supporting the biological data. The combined results demonstrate that PrrAB contributes to optimal growth during and after hypoxic stress. We recently reported that the Δ*prrAB* mutant is hypersensitive to hypoxia during growth in low-ammonium medium [19]. Our current data suggest that the hypoxia growth defect exhibited by the Δ*prrAB* mutant is likely not medium-specific, but rather a global consequence of differential regulation of respiratory and/or the *dosR* regulon genes. Bacterial cytochrome *bd* oxidases are relatively insensitive to cyanide inhibition compared to the cytochrome c oxidase respiratory branch [53–55]. Growth of the Δ*prrAB* mutant in the presence of 1 mM potassium cyanide was similar to *M. smegmatis cydA* mutant growth under similar conditions [24]. Our data demonstrates that the *M. smegmatis* PrrAB TCS controls expression of aerobic and microaerophilic respiratory genes. Notably, to date, a master transcriptional regulator of respiratory systems in *M. tuberculosis* has not been discovered.

We found increased expression of the F_1_F_0_ ATP synthase genes, including *atpA, atpD, atpF, atpG,* and *atpH,* in the Δ*prrAB* mutant strain compared to WT (Fig. 7a; Additional file 1: Figure S13 and Additional file 2), leading us to hypothesize that ATP levels would be elevated in the Δ*prrAB* mutant. Conversely, ATP levels were lower in Δ*prrAB* mutant strain compared to the WT and complementation strains (Fig. 7c). Induction of *atp* genes in the Δ*prrAB* mutant may indicate a compensatory measure to maintain ATP homeostasis due to repression of the *bc*_1_*-aa*_3_ terminal respiratory complex (except *ctaB*) and hence, disruption of the transmembrane proton gradient.

Via comprehensive transcriptomics analyses, we demonstrated that PrrAB regulates expression of genes involved in respiration, environmental adaptation, ion homeostasis, oxidoreductase activity, and metabolism in *M. smegmatis*. The inability to induce transcription of the *cydA, cydB, cydD, dosR1*, and *dosR2* genes likely led the Δ*prrAB* mutant to grow poorly after 24 h hypoxia exposure. An important goal of our RNA-seq study was to provide insight into the essential nature of PrrAB in *M. tuberculosis* using an *M. smegmatis* Δ*prrAB* mutant as a surrogate model while recognizing differences in their natural environmental niches, pathogenic potential, and genetic composition. From a therapeutic perspective, PrrAB could influence the sensitivity of *M. tuberculosis* to Q203 and/or bedaquiline by controlling expression of cytochrome *bd* oxidase, cytochrome c *bc*_1_ oxidase, and ATP synthase genes. Furthermore, it remains unknown whether diarylthiazoles directly target PrrB [17] or whether the *prrB* mutations associated with diarylthiazole resistance are compensatory in nature. Taken together, our study provides seminal information regarding the mycobacterial PrrAB TCS regulon as well as a powerful surrogate platform for in-depth investigations of this essential TCS in *M. tuberculosis*.

## Conclusions

We used RNA-seq-based transcriptomics as an experimental platform to provide insights into the essential *M. tuberculosis prrAB* TCS using an *M. smegmatis* Δ*prrAB* mutant as a genetic surrogate. In *M. smegmatis*, PrrAB regulates high-affinity respiratory systems, intracellular redox and ATP balance, and the *dosR* TCS response regulator genes, all of which promote infectious processes in *M. tuberculosis*. Using these results, we may be able to exploit diarylthiazole compounds that putatively target the PrrB histidine kinase as synergistic therapies with bedaquiline. These results are informing the basis of *prrAB* essentiality in *M. tuberculosis* and advancing our understanding of regulatory systems that control metabolic, respiration, energy-generating, and dormancy pathways in mycobacteria. Exploitation of PrrAB as a drug target will advance the discovery and development of novel therapeutics to combat the global tuberculosis epidemic.

## Methods

### Bacterial strains and culture conditions

Genetic construction of the *M. smegmatis* FDL10 Δ*prrAB* deletion mutant and the FDL15 complementation strain was previously described [19]. All *M. smegmatis* strains (mc^2^155, FDL10, and FDL15) were routinely cultured in Middlebrook 7H9 broth (pH 6.8) supplemented with 10% albumin-dextrose-saline (ADS), 0.2% glycerol (v/v), and 0.05% Tween 80 (v/v), herein referred to as M7H9. *M. smegmatis* was incubated on Middlebrook 7H10 agar supplemented with 10% ADS and 0.5% glycerol, herein referred to as M7H10 agar, for CFU/ml enumeration.

### Hypoxic growth conditions

*M. smegmatis* strains were initially cultured aerobically in M7H9 medium at 37 °C, 100 rpm to an OD_600_ ~ 0.6. Cells were diluted into fresh, pre-warmed M7H9 to an OD_600_ ~ 0.05, serially diluted in PBS (pH 7.4), and spot-plated onto M7H10 agar. The plates were transferred to a GasPak chamber containing two anaerobic GasPak sachets (Beckon Dickinson, Franklin Lakes, NJ, USA), sealed, and incubated at 37 °C for 24 h after the onset of hypoxia (~ 6 h), as indicated by decolorization of an oxygen indicator tablet included with the sachet. Plates were then incubated aerobically for an additional 48 h to allow colony outgrowth. Control plates were cultured under aerobic conditions for 48 h prior to counting and documenting colonies. Colonies were visualized using a dissecting microscope (Stereomaster, Fisher Scientific). All experiments were performed in triplicate.

### Cyanide inhibition assays

*M. smegmatis* strains were grown in the presence of potassium cyanide (KCN) as described by [24] with modifications. Briefly, cultures were inoculated into prewarmed M7H9 broth to an OD_600_ ~ 0.05 and incubated at 37 °C, 100 rpm for 30 min. KCN, prepared in M7H9 broth, was then added to a final concentration of 1 mM and growth was allowed to resume. Negative control cultures using M7H9 broth without KCN addition were performed concurrently. Cultures were grown for 5 d with samples collected at 24 h intervals for OD_600_ measurements and CFU quantitation on M7H10 agar. All experiments were performed in triplicate.

### ATP assays

*M. smegmatis* strains were cultured in M7H9 broth at 37 °C, 100 rpm. Cultures were sampled in 100 μl aliquots upon reaching an OD_600_ ~ 0.6, flash-frozen in a dry ice-ethanol bath, and stored at − 70 °C for 7 d. Cells were thawed at room temperature and ATP quantification was performed using the BacTiter-Glo kit (Promega, Madison, WI, USA). Fifty μl of cells were mixed with equal volumes of BacTiter-Glo reagent in opaque 96-well plates and incubated at room temperature for 5 min. ATP standard curves were included in the same plate. Relative luminescence was measured in a SpectraMax M5 plate reader (Molecular Devices, San Jose, CA, USA). To assess lysis efficiency, viability of all samples was confirmed after both freeze-thaw and processing in the BacTiter-Glo reagent by plating serial dilutions onto M7H10 agar followed by incubation at 37 °C for 48–72 h. Lysis efficiencies collected from three independent cultures of mc^2^155, FDL10, and FDL15 were 99.97% (± 0.03), 99.99% (± 0.04), and 99.99% (± 0.02), respectively. Cell viability was quantified for each sample at the time of harvest by plating serial dilutions onto M7H10 agar followed by incubation at 37 °C for 48 h before enumerating CFU/ml. Samples for extracellular ATP measurement were collected as described by Hirokana et al. [56]. Briefly, cells were harvested by centrifugation at 10,621 x *g* for 2 min at 4 °C. The supernatant was clarified via 0.22 μm filtration, and aliquots (100 μl) were flash-frozen in a dry ice-ethanol bath and stored at − 70 °C until further use. After thawing, ATP was measured using the BacTiter-Glo kit, as described above. Filtered supernatants were spot plated onto M7H10 agar and incubated at 37 °C for 3 d to verify lack of contaminating cells. All strains were analyzed in triplicate with two technical replicates each.

### RNA isolation

For aerobic cultures, *M. smegmatis* strains mc^2^155, FDL10, and FDL15 were grown in 30 ml M7H9 at 37 °C, 100 rpm until mid-logarithmic phase (OD_600_ ~ 0.6). For hypoxic cultures, *M. smegmatis* strains were first grown (OD_600_ ~ 0.6) aerobically in M7H9. Each culture (15 ml) was then transferred a fresh tube, and methylene blue (1.5 μg/ml, final concentration) was added as an indicator of O_2_ depletion. Cultures were incubated in a sealed GasPak chamber containing two anaerobic sachets (Beckon Dickinson, Franklin Lakes, NJ, USA) for 24 h post-decolorization of the methylene blue in the media. Culture aliquots (15 ml) were harvested by centrifugation at 3441 x *g* for 10 min at 4 °C. The supernatant was discarded, and the cell pellet was resuspended in 1 ml TRIzol (Invitrogen), transferred to 2 ml screw cap tubes containing 500 mg of zirconia-silicate beads (0.1–0.15 mm), and placed on ice. Cells were mechanically disrupted 3X by bead beating (BioSpec Products) at the highest setting for 40 s and incubated on ice for at least 1 min between disruptions. The cell lysates were incubated at room temperature for 5 min, centrifuged at 13,000 x *g* for 1 min to separate cell debris, and the supernatant was transferred to a new microcentrifuge tube. Chloroform (200 μl) was added, and samples were vortexed for 15 s followed by 5 min incubation at 4 °C. The homogenate was centrifuged at 13,000 x *g* for 15 min at 4 °C and the upper, aqueous phase was transferred to a new microcentrifuge tube. RNA was precipitated with 500 μl isopropanol overnight at 4 °C. Total RNA was pelleted by centrifugation at 13,000 x *g* for 15 min at 4 °C, and the supernatant was discarded. RNA pellets were washed 2X with 70% ethanol and centrifuged at 13,000 x *g* for 5 min at 4 °C between washes. After evaporation of residual ethanol by air-drying, total RNA was resuspended in 100 μl nuclease-free H_2_O. Total RNA (10 μg) was treated with TURBO-DNase (Invitrogen, Carlsbad, CA) for 20 min at 37 °C to degrade residual genomic DNA. RNA samples were purified using the RNeasy Mini Kit (Qiagen, Germany) and eluted in 50 μl nuclease-free H_2_O. RNA yields were quantified by Nanodrop (Thermo Scientific, Waltham, MA), and quality was assessed by agarose gel electrophoresis and a 2100 Bioanalyzer (Agilent, Santa Clara, CA). RNA (250 ng) was subjected to PCR using primers directed at the *16S* rRNA gene to confirm lack of residual genomic DNA.

### RNA-seq library preparation

cDNA was generated from RNA using the Nugen Ovation RNA-seq System via single primer isothermal amplification and automated on the BRAVO NGS liquid handler (Agilent, Santa Clara, CA, USA). cDNA was quantified on the Nanodrop (Thermo Fisher Scientific) and was sheared to approximately 300 bp fragments using the Covaris M220 ultrasonicator. Libraries were generated using the Kapa Biosystem’s library preparation kit (Kapa Biosystems, Wilmington, MA, USA). Fragments were end-repaired and A-tailed and individual indexes and adapters (Bioo, catalogue #520999) were ligated on each separate sample. The adapter-ligated molecules were cleaned using AMPure beads (Agencourt Bioscience/Beckman Coulter, La Jolla, CA, USA), and amplified with Kapa’s HIFI enzyme (Kapa Biosystems, Wilmington, MA, USA). Each library was then analyzed for fragment size on an Agilent Tapestation and quantified by qPCR (KAPA Library Quantification Kit, Kapa Biosystems, Wilmington, MA, USA) using Quantstudio 5 (Thermo Fisher Scientific) prior to multiplex pooling.

### Sequencing and data processing

Sequencing was performed on a 1 × 75 bp flow cell using the NextSeq500 platform (Illumina) at the ASU Genomics Core facility. The total number of 101,054,986 Illumina NextSeq500 paired-end reads were generated from nine RNA samples (i.e., triplicates for each strain). The total number of reads generated for each sample ranged from 7,729,602 to 14,771,490. RNA-seq reads for each sample were quality checked using FastQC v 0.10.1 and aligned to the *Mycolicibacterium smegmatis* MC2155 assembly obtained from NCBI (https://www.ncbi.nlm.nih.gov/assembly/GCF_000015005.1/) using STAR v2.5.1b. Cufflinks v2.2.1 was used to report FPKM (Fragments Per Kilobase of transcript per Million mapped reads) values and the read counts. As a quality check for the biological replicates, overall similarity of gene expression profiles were then assessed by MDS, in which distances correspond to leading log-fold changes between samples. The MDS analysis demarcated clearly one of the three mc^2^155 samples as an outlier that did not cluster with the other two mc^2^155 samples and the three FDL15 samples (See Additional file 1: Figure S3), and the sample was thus excluded from further analysis. Average genome-wide expression (FPKM) was 6.76 for the WT strain, 5.88 for the Δ*prrAB* mutant, and 6.38 for the complementation strain.

### Bioinformatics analysis

Differential expression analysis was performed with EdgeR package from Bioconductor v3.2 in R 3.2.3. EdgeR applied an overdispersed Poisson model to account for variance among biological replicates. Empirical Bayes tagwise dispersions were also estimated to moderate the overdispersion across transcripts. Then, a negative binomial generalized log-linear model was fit to the read counts for each gene for all comparison pairs. For each pairwise comparison, genes with *p* values < 0.05 were considered significant and log_2_-fold changes of expression between conditions (logFC) were reported. False discovery rate was calculated following the Benjamini and Hochberg procedure [57], the expected proportion of false discoveries amongst the rejected hypotheses.

Principal component analysis (PCA) was done on the scaled data using the prcomp function in R. Clustering analysis was done using Cluster 3.0 software, in which normalized expression (FPKM + 1) values were log_2_ transformed and grouped using uncentered Pearson’s correlation distance and average linkage hierarchal clustering [58]. Data matrices and tree dendrograms were visualized in Java TreeView. Gene ontology (GO) term enrichment, KEGG pathways, and statistical analyses of differentially expressed genes were performed using the DAVID functional annotation tool (https://david.ncifcrf.gov/summary.jsp). Clusters of orthologous groups (COGs) were obtained by querying DEGs (*q* < 0.05) against the eggNOG Mapper database (http://eggnogdb.embl.de/#/app/emapper).

### Quantitative RT-PCR (qRT-PCR)

cDNA libraries from each RNA sample (described above) were generated by reverse transcription of 1 μg total RNA using the iScript cDNA Synthesis Kit (Bio-Rad, Hercules, CA, USA), according to the manufacturer’s instructions. Primer efficiency was validated against 10-fold dilution standard curves using a cutoff criterion for acceptable efficiency of 90–110% and coefficient of determination (*R*^*2*^) ≥ 0.997. Relative gene expression was calculated using the 2^-ΔCt^ or 2^-ΔΔCt^ method [59], as indicated, and using the *16S* gene as an internal normalization reference. The primers used for qRT-PCR are described in Table S1 (See Additional file 1).

### Phylogenetic analyses

The *M. smegmatis* mc^2^155 PrrA and PrrB sequences were separately queried in BLASTp (https://blast.ncbi.nlm.nih.gov/Blast.cgi) against all Mycobacteriacea (taxid: 1762). Sequences corresponding to the revised mycobacterial phylogenetic clade classification [20] were selected for further analysis. When multiple hits were returned from the same species, those corresponding to the lowest E-value were selected for alignment. Compiled PrrA and PrrB sequences were separately aligned in MEGA 7 (https://www.megasoftware.net/) using default MUSCLE algorithms. Maximum-likelihood phylogenetic trees were generated in MEGA 7 and visualized by iTOL [60].

### Statistical analyses

We used one-way ANOVA to assess significant differences in cell viability, qRT-PCR gene expression, and ATP quantification assays. Statistical analyses were performed using GraphPad Prism 7 (GraphPad Software, San Diego, CA) and *p-*values of < 0.05 were considered statistically significant. For volcano plot data, the -log_10_ *p*-value of each DEG was plotted against the ratio of the mean log_2_-fold change of each differential expressed gene between FDL10 vs. mc^2^155 or FDL10 vs. FDL15.

## Supplementary information

**Additional file 1.** Supplemental figures.

**Additional file 2.** *M. smegmatis* DEG data sets.

**Additional file 3.** DAVID gene ontology results from significant DEGs.

**Additional file 4.** DosR regulon DEG and comparison of genes betweeen *M. smegmatis* and *M. tuberculosis*.

**Additional file 5.** RNA Bioanalyzer results (RIN numbers and electrophoretic traces).

**Additional file 6.** DAVID gene ontology results from overlapping DEGs between mc^2^155 vs. FDL10 and FDL15 vs. FDL10 data sets.

## Data Availability

The raw Illumina paired-end sequence data for the RNA-seq studies performed in this article are available at the NCBI Sequence Read Archive (SRA) under the BioProject number PRJNA532282 under accession numbers SAMN11393348, SAMN11393349, and SAMN11393350. The assembled genome sequence for *Mycolicibacterium smegmatis* MC2 155 can be found in the GenBank database under assembly accession GCA_000015005.1.

## Abbreviations

ADS: Albumin-dextrose-saline
COG: Clusters of orthologous groups
DEG: Differentially expressed gene
FPKM: Fragments per kilobase of transcript per million mapped reads
GO: Gene ontology
KCN: Potassium cyanide
logFC: log_2_ fold change
M7H9: Middlebrook 7H9
MDS: Multidimensional scaling
PCA: Principal component analysis
qRT-PCR: Quantitative reverse transcriptase PCR
TCS: Two-component system
WT: Wild-type
