# Correction to: Comparative transcriptomics reveals PrrABmediated control of metabolic, respiration, energy-generating, and dormancy pathways in Mycobacterium smegmatis

**DOI:** 10.1186/s12864-019-6419-1

**Published:** 2019-12-31

**Authors:** Jason D. Maarsingh, Shanshan Yang, Jin G. Park, Shelley E. Haydel

**Affiliations:** 1 School of Life Sciences, Arizona State University, Tempe, AZ USA; 2 Department of Obstetrics and Gynecology, College of Medicine-Phoenix, University of Arizona, Phoenix, AZ USA; 3 Bioinformatics Core, Knowledge Enterprise Development, Arizona State University, Tempe, AZ USA; 4 The Biodesign Institute Virginia G. Piper Center for Personalized Diagnostics, Arizona State University, Tempe, AZ USA; 5 The Biodesign Institute Center for Immunotherapy, Vaccines and Virotherapy, Arizona State University, Tempe, AZ USA

**Correction to: BMC Genomics**

**https://doi.org/10.1186/s12864-019-6105-3**

Following the publication of the original article [1], the authors reported an error in Fig. 1 of the PDF version of their article. Due to a typesetting mistake, a previous version of the figure was placed in the PDF, which therefore did not match the correct Fig. 2 given in the HTML version.

The incorrect figure was:

**Fig. 2:**
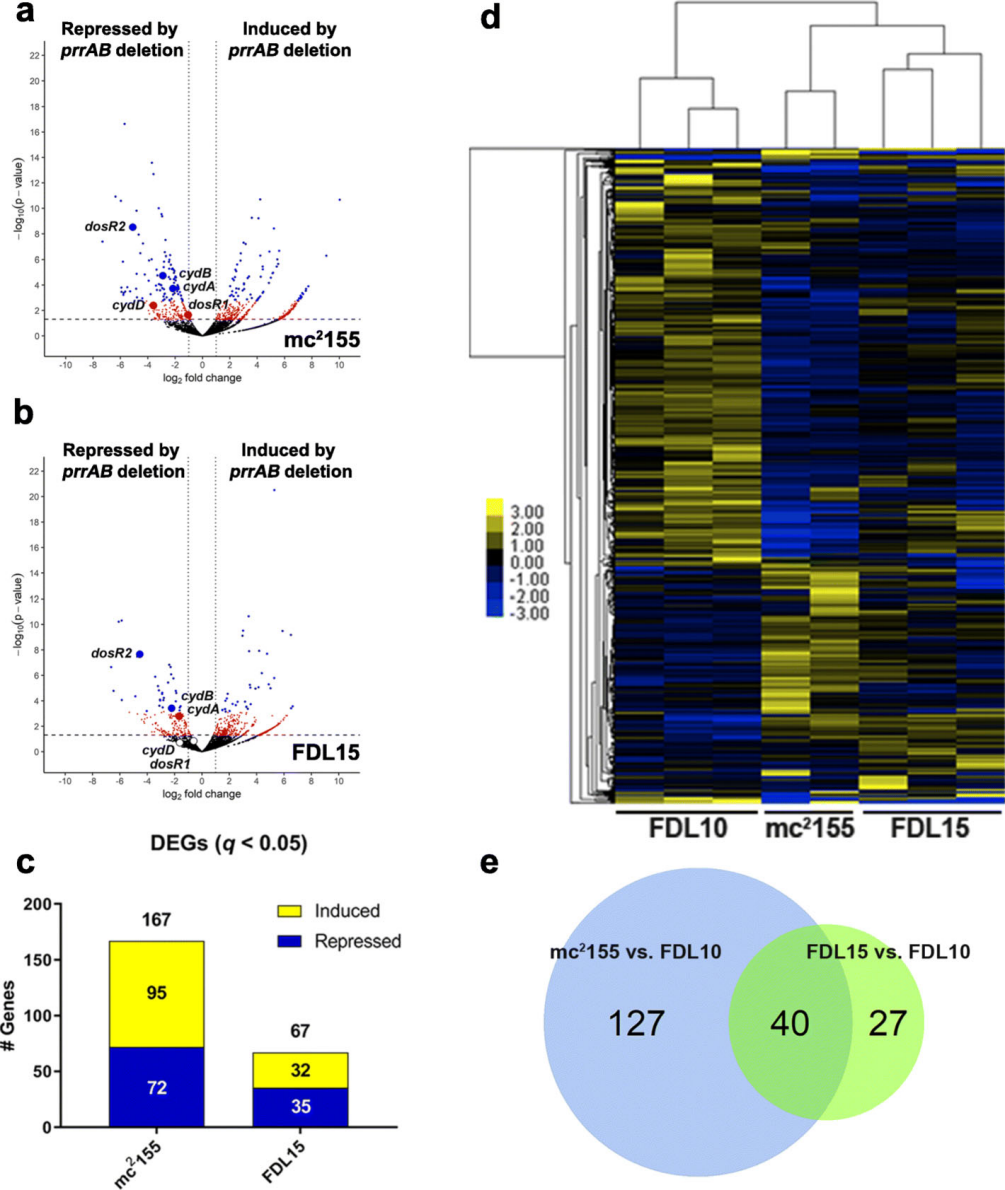
Global DEG profiles (q < 0.05) between the mc2 155 vs. FDL10 and FDL15 vs. FDL10 RNA-seq comparisons. Volcano plots of (a) FDL10 vs. mc2 155 and (b) FDL10 vs. FDL15 group comparisons with red and blue dots representing differentially-expressed genes with *p* < 0.05 and q < 0.05, respectively. The horizontal hatched line indicates *p* = 0.05 threshold, while the left and right vertical dotted lines indicate log2 fold change of − 1 and + 1, respectively. c Repressed (blue) and induced (yellow) DEGs (q < 0.05) in mc2 155 (WT) and FDL15 (prrAB complementation strain) compared to the FDL10 ΔprrAB mutant. d Average hierarchical clustering (FPKM + 1) of individual RNA-seq sample replicates. e Venn diagrams indicating 40 overlapping DEGs (q < 0.05) between mc2 155 vs. FDL10 (WT vs. ΔprrAB mutant) and FDL15 vs. FDL10 (prrAB complementation strain vs. ΔprrAB mutant) strain comparisons

The correct figure is:

**Fig. 2:**
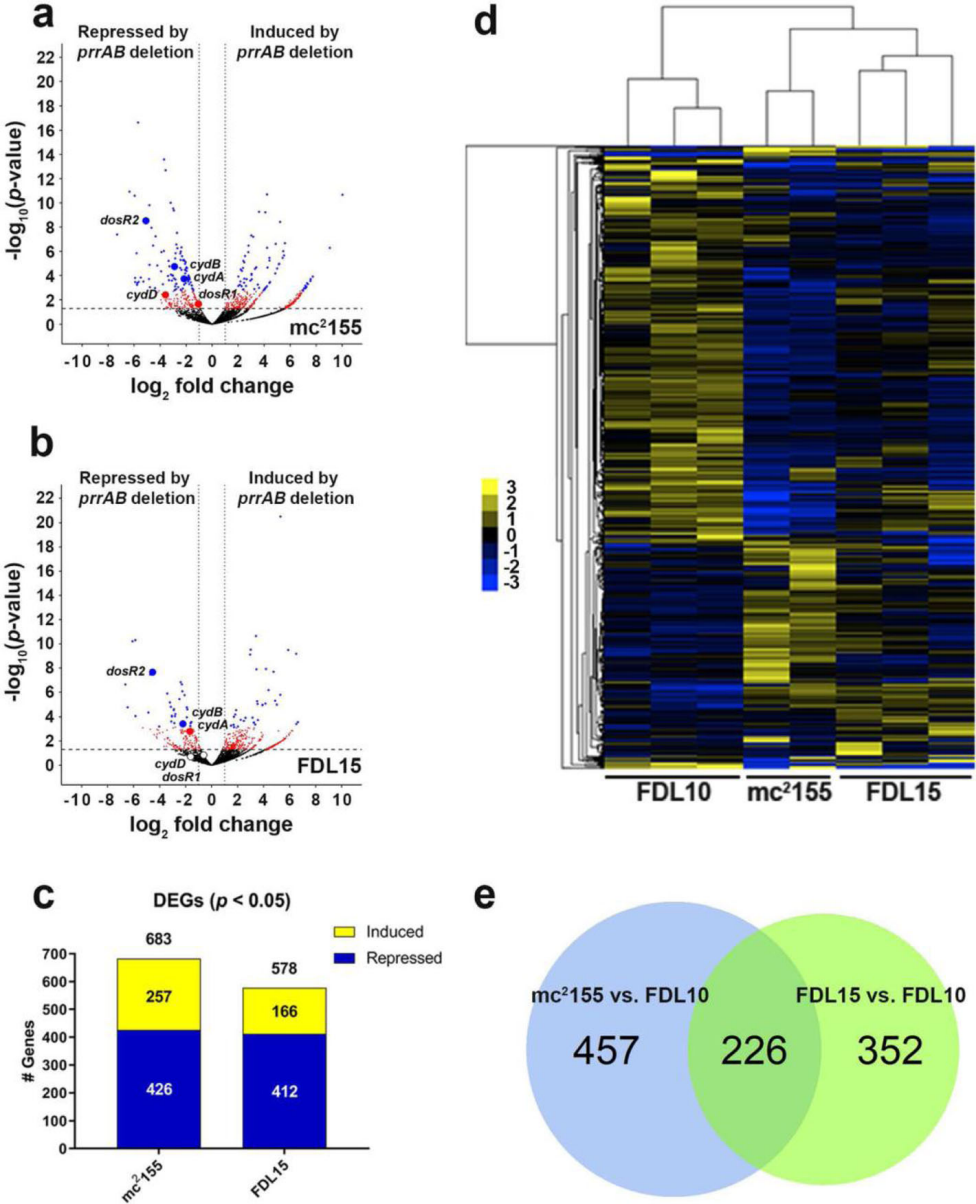
Global DEG profiles (q < 0.05) between the mc2 155 vs. FDL10 and FDL15 vs. FDL10 RNA-seq comparisons. Volcano plots of (a) FDL10 vs. mc2 155 and (b) FDL10 vs. FDL15 group comparisons with red and blue dots representing differentially-expressed genes with *p* < 0.05 and q < 0.05, respectively. The horizontal hatched line indicates *p* = 0.05 threshold, while the left and right vertical dotted lines indicate log2 fold change of − 1 and + 1, respectively. c Repressed (blue) and induced (yellow) DEGs (q < 0.05) in mc2 155 (WT) and FDL15 (prrAB complementation strain) compared to the FDL10 ΔprrAB mutant. d Average hierarchical clustering (FPKM + 1) of individual RNA-seq sample replicates. e Venn diagrams indicating 40 overlapping DEGs (q < 0.05) between mc2 155 vs. FDL10 (WT vs. ΔprrAB mutant) and FDL15 vs. FDL10 (prrAB complementation strain vs. ΔprrAB mutant) strain comparisons

The original article has been corrected.
