# Supplementary material for: Comparative transcriptomics reveals PrrAB-mediated control of metabolic, respiration, energy-generating, and dormancy pathways in Mycobacterium smegmatis
Source: BMC Genomics. 2019 Dec 7;20:942. doi: 10.1186/s12864-019-6105-3 (PMC6898941; doi:10.1186/s12864-019-6105-3)
Supplement: Supplementary file 1 — Additional file 1. Supplemental figures. [file 12864_2019_6105_MOESM1_ESM.docx]

**Comparative transcriptomics reveals PrrAB-mediated control of metabolic, respiration, energy-generating, and dormancy pathways in *Mycobacterium smegmatis***

**JASON D. MAARSINGH^1^, SHANSHAN YANG^2^, JIN G. PARK^3^, and SHELLEY E. HAYDEL^1,4^***

*^1^School of Life Sciences, Arizona State University, Tempe AZ, USA*

^2^*Bioinformatics Core, Knowledge Enterprise Development, Arizona State University, Tempe AZ, USA*

*^3^The Biodesign Institute Virginia G. Piper Center for Personalized Diagnostics, Arizona State University, Tempe AZ, USA*

*^4^The Biodesign Institute Center for Immunotherapy, Vaccines and Virotherapy, Arizona State University, Tempe AZ, USA*

**Additional File 1: Supplemental table and figures**

**Table S1.** qRT-PCR primers used for qRT-PCR in this study.

| **Gene** | **Forward primer (5’ 🡪 3’)** | **Reverse primer (5’ 🡪 3’)** |
| --- | --- | --- |
| *16S* | CCTATCAGCTTGTTGGTGG | GTGCAATATTCCCCACTG |
| *atpC* | TGGCTGATCTGAACGTCG | CAGGATGCCGATCTCACC |
| *atpI* | CTTGCTGGTCGTTTGCG | CGATGCCGAAGAACATGC |
| *atpH* | GGACCCTGATGCGTAACC | TCCTCGGTGACCTTCTCC |
| *cydA* | CACTTCATATTTGTCCCGCTG | AGTACTCGCTCCAGTTCATG |
| *cydB* | CCCTACACCCTCAAGATCATG | TTGCTGAACACCCAGTACG |
| *cydD* | TATTTCACGGGCTACCTGC | ATCAGCACCATGAAGATCGG |
| *dosR1* | ACCTGTTGTCGCACATG | TCCTTGACCACGTATCCGCTCG |
| *dosR2* | TCTGCGTTGTCTGATGCTC | TGCCCTTGATGTCCTTGATG |
| *MSMEG 0911* | CTGGCCTACAACTGCTCG | TGAACTGGAACTTGAAGCCC |
| *MSMEG 3948* | GTCTCGTCATCGGTGTGC | GTGGTGGATCTTCATCAGG |
| *MSMEG 4757* | TGTCATGCAGTCGTATGTGG | TGCCGAGCTTGATCTTGTC |
| *MSMEG 5242* | GAACAGCTCTCCGTGCTC | GTGGAGTACCTGCCTCAG |
| *MSMEG 6242* | AGTGCGACAGCATCATCTC | TCTGCTTGTTGGTGGACTT |
| *MSMEG 6935* | TCATGTCGTATCAGCGTGAG | GTGAGCCGAGGAAGTACAG |


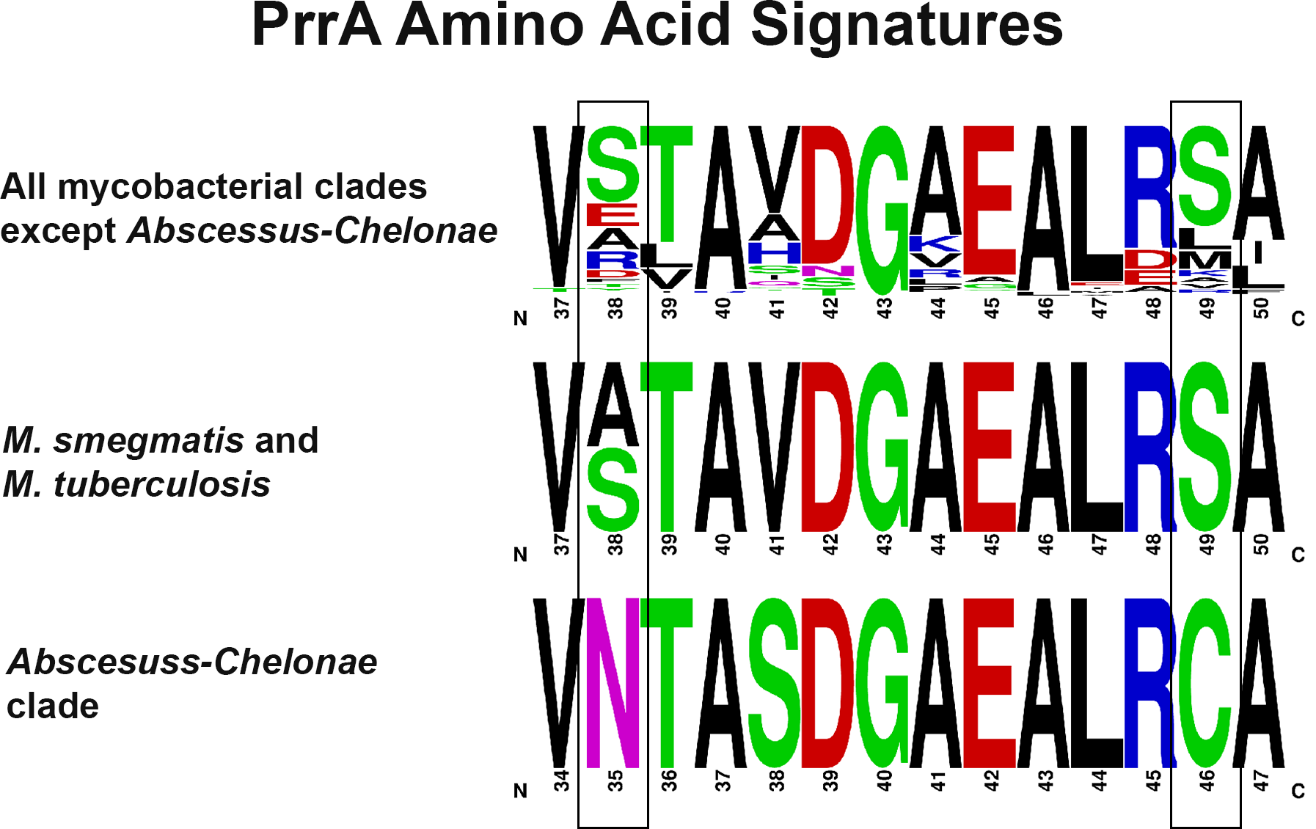


**Figure S1.** Members of the mycobacterial *Abscessus-Chelonae* clade harbor unique PrrA amino acid “signatures”. Boxed residues correspond to amino acid residues only found in mycobacterial species belonging to the *Abscessus-Chelonae* clade (bottom row) compared to all other mycobacterial clades (top row) or *M. smegmatis* mc^2^155 and *M. tuberculosis* H37Rv (middle row). Numerical system below single-letter amino acid codes correspond to the residue position in *M. smegmatis* (top and middle rows) or *M. abscessus* (bottom row). Left box corresponds to PrrA residue S38 of *M. smegmatis* (top and middle rows) and N35 of *M. abscessus*. Right box corresponds to PrrA residue S49 of *M. smegmatis* (top and middle rows) and C46 of *M. abscessus*.


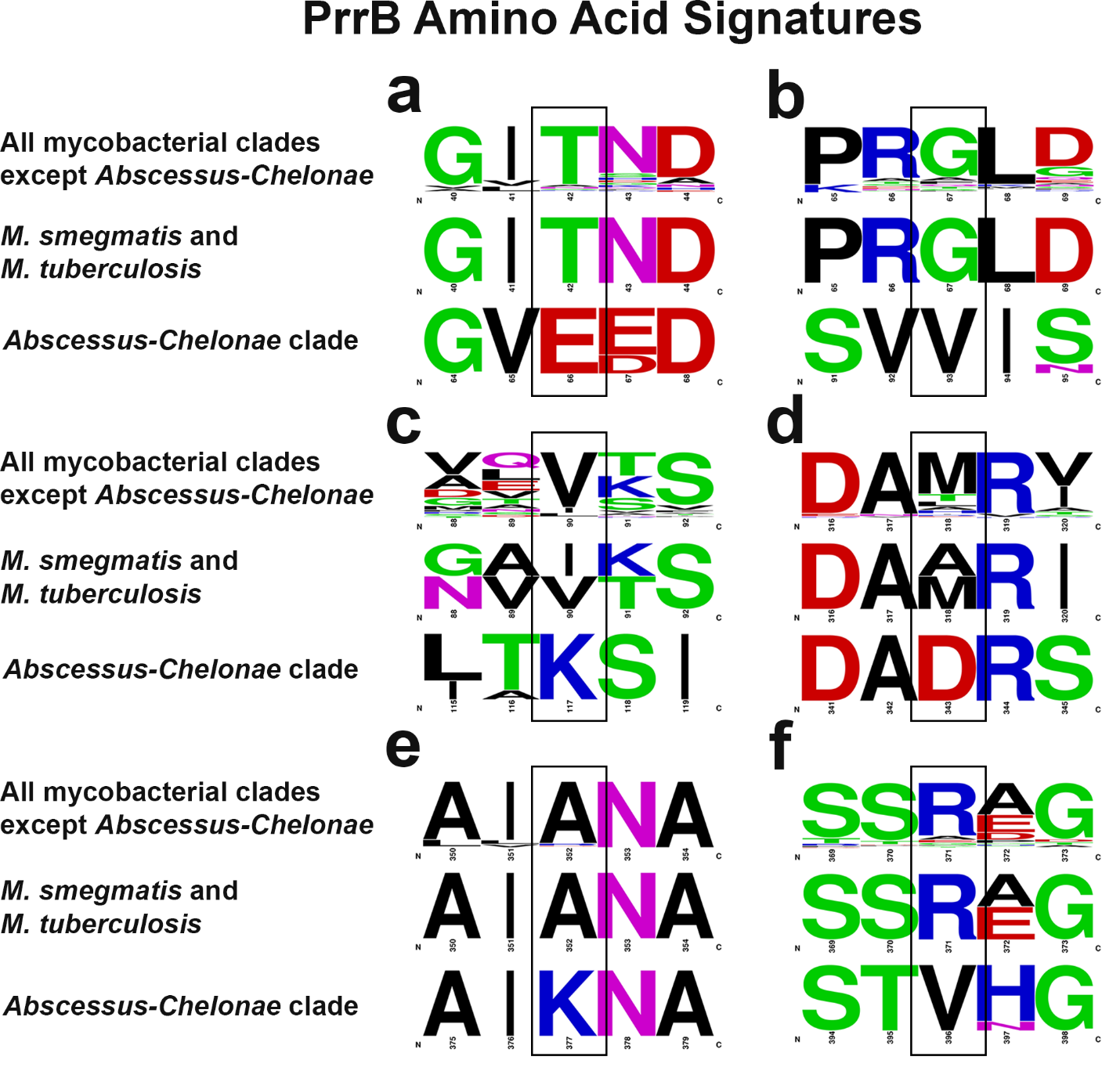


**Figure S2.** Members of the mycobacterial *Abscessus-Chelonae* clade harbor unique PrrB amino acid “signatures”. Boxed residues correspond to amino acid residues only found in mycobacterial species belonging to the *Abscessus-Chelonae* clade (bottom row) compared to all other mycobacterial clades (top row) or *M. smegmatis* mc^2^155 and *M. tuberculosis* H37Rv (middle row). Numerical system below single-letter amino acid codes correspond to the residue position in *M. smegmatis* (top and middle rows) or *M. abscessus* (bottom row). (**a**) PrrB residue T42 of *M. smegmatis* (top and middle rows) and E66 of *M. abscessus*. (**b**) PrrB residue G67 of *M. smegmatis* (top and middle rows) and V93 of *M. abscessus*. (**c**) PrrB residue V90 of *M. smegmatis* (top and middle rows) and 117 of *M. abscessus*. (**d**) PrrB residue M318 of *M. smegmatis* (top and middle rows) and D343 of *M. abscessus*. (**e**) PrrB residue A352 of *M. smegmatis* (top and middle rows) and K377 of *M. abscessus*. (**f**) PrrB residue R371 of *M. smegmatis* (top and middle rows) and V396 of *M. abscessus*.


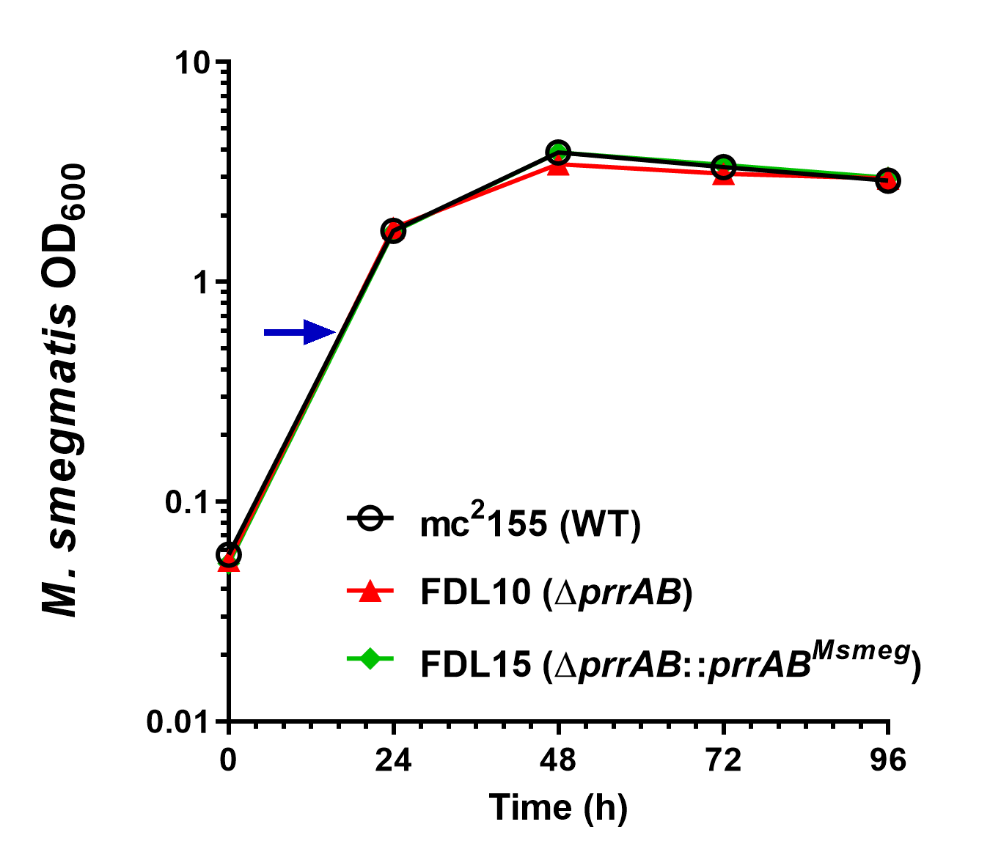


**Figure S3:** *M. smegmatis* growth characteristics in M7H9 broth. Optical density (OD_600_) of mc^2^155 (open circles), FDL10 (red triangles), and FDL15 (green diamonds). The blue arrow shows the OD_600_ (~0.6) when cultures were collected for RNA isolation. Values represent the mean ±SEM of data collected from three independent cultures.


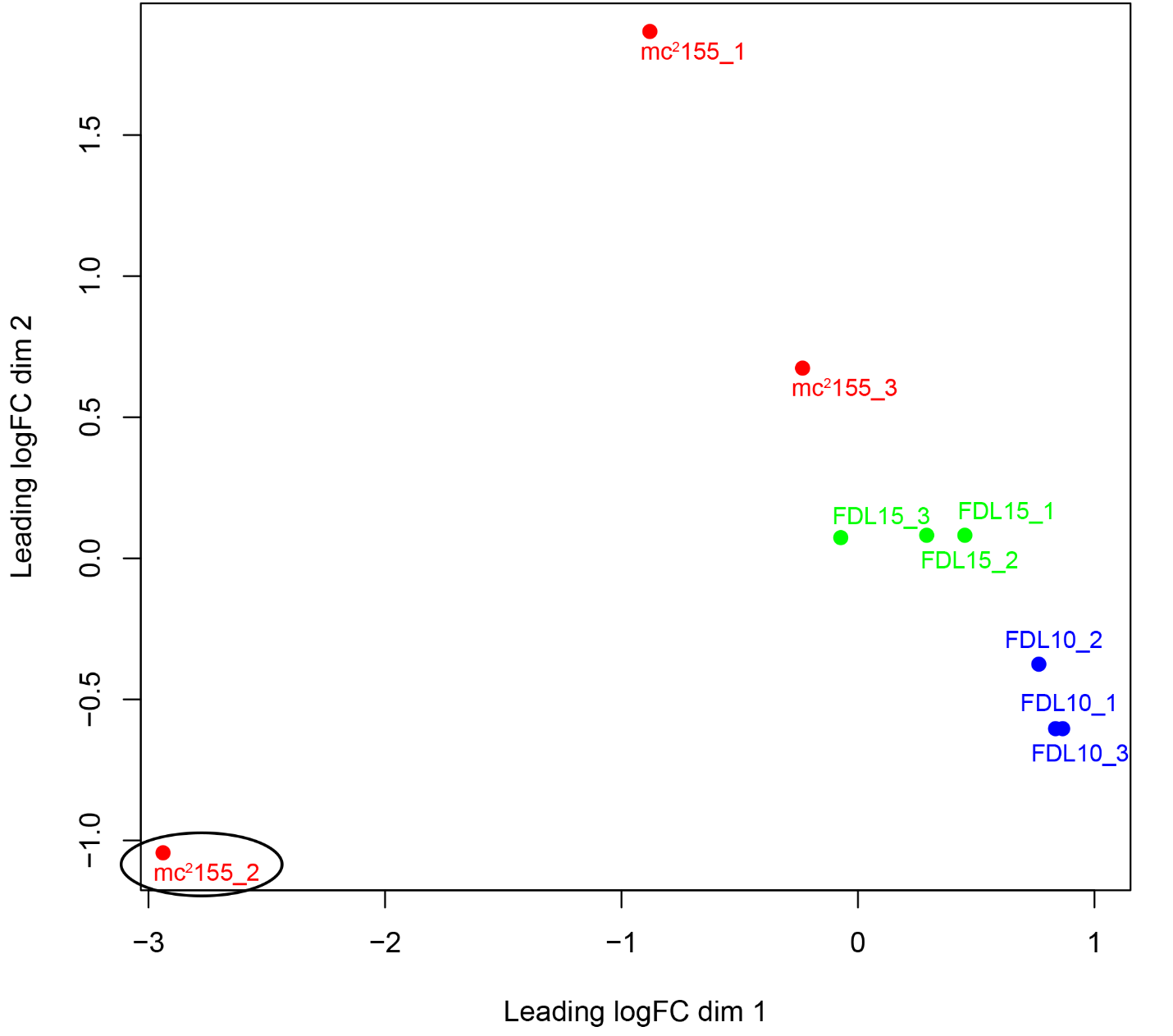


**Figure S4.** Multidimensional scaling (MDS) plot of triplicate *M. smegmatis* RNA-seq samples. Given the MDS-based spatial separation of the mc^2^155_2 sample (circled in the bottom-left corner of plot) from other mc^2^155 replicates, the mc^2^155_2 sample was deemed an outlier and removed from differential expression analysis.


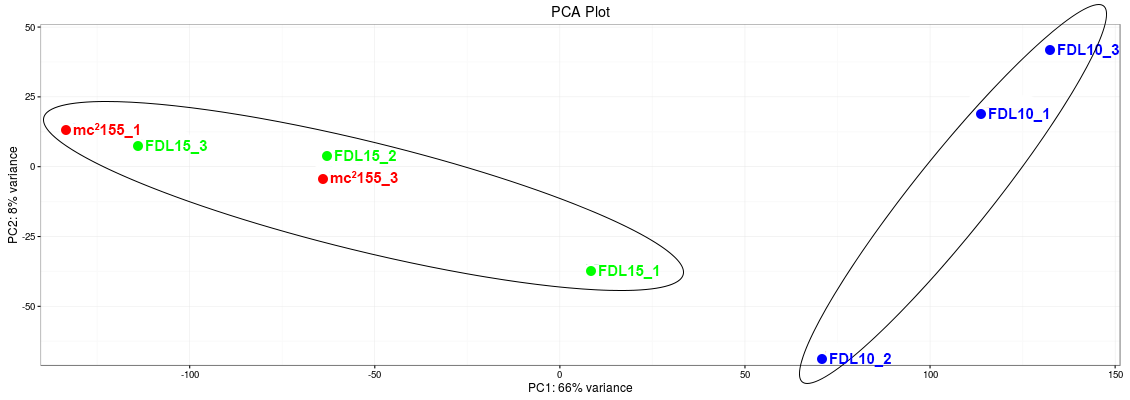


**Figure S5.** Principal component analysis (PCA) of *M. smegmatis* strains used for RNA-seq DEG analyses.


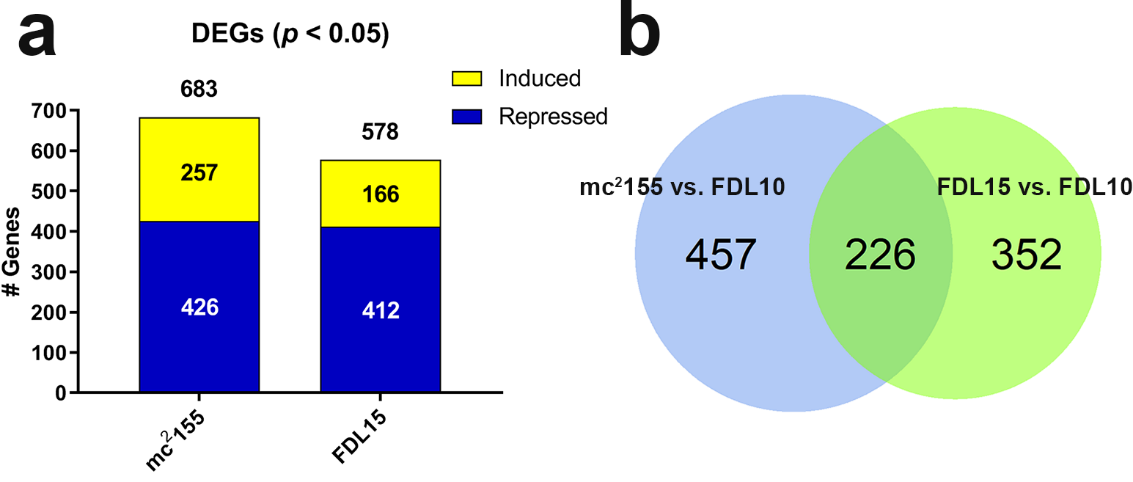


**Figure S6.** Global expression profile of DEGs (*p* <0.05). (**a**) Total DEGs (*p* < 0.05) induced (yellow) or repressed (blue) by PrrAB in mc^2^155 (WT) and FDL15 (Δ*prrAB* complementation) backgrounds (RNA-seq pair-wise comparisons to the Δ*prrAB* mutant). (**b**) Venn diagrams of DEGs (*p* < 0.05) demonstrating that 226 DEGs (*p* < 0.05) overlapped between RNA-seq pair-wise comparisons.


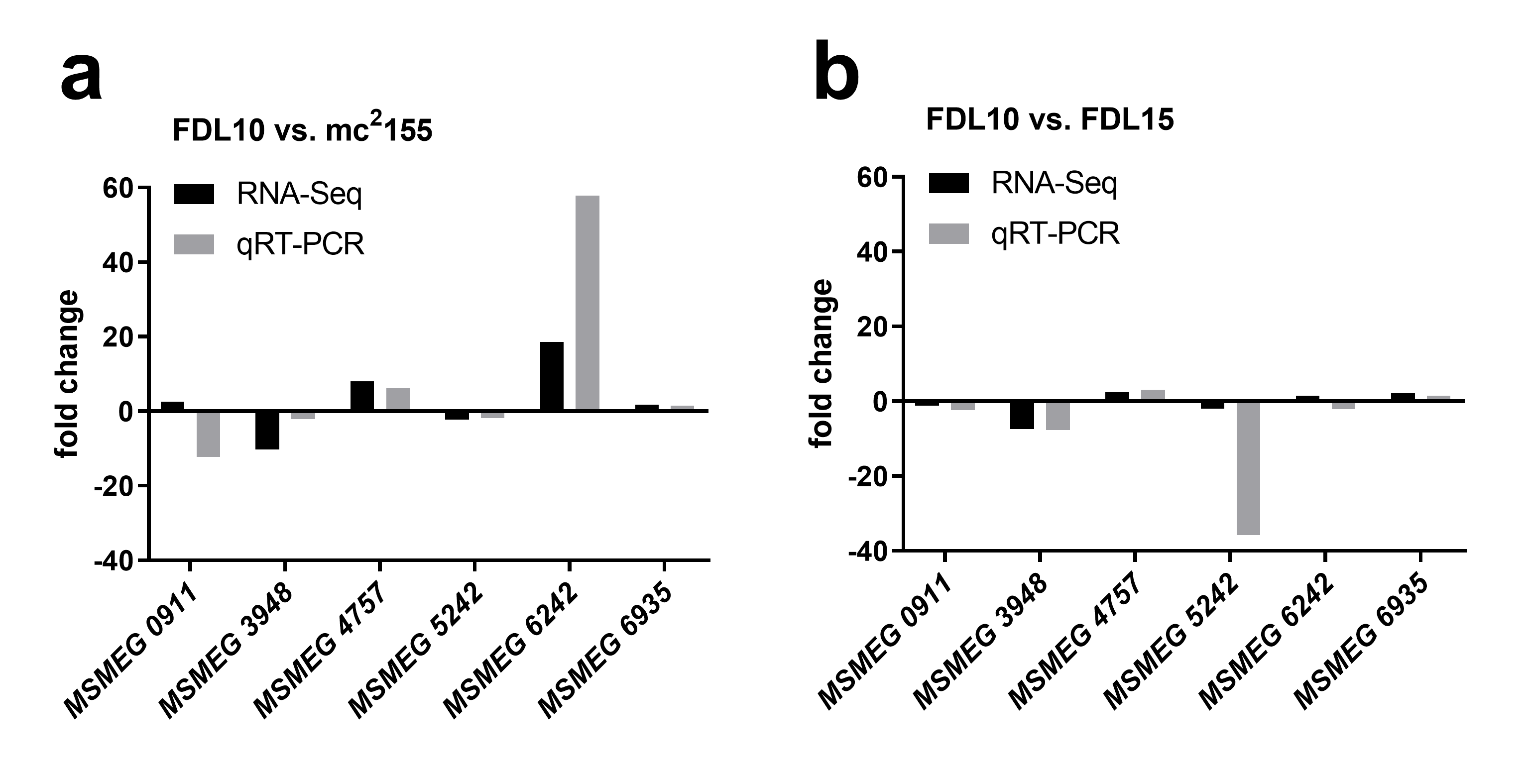


**Figure S7.** qRT-PCR verification of six randomly selected genes from the RNA-seq (**a**) FDL10 vs. mc^2^155 and (**b**) FDL10 vs. FDL15 comparisons. All qRT-PCR measurements were performed from the same RNA samples used for RNA-seq analyses and each gene was tested in triplicate. Absolute fold change ratios were calculated using the 2­^-ΔΔCt^ method [1]. Values represent absolute fold change for both qRT-PCR and RNA-seq data. DEGs with *q* < 0.05: *MSMEG 3948, MSMEG 4757, MSMEG 6242*. DEGs with *p >* 0.05 (not significant): *MSMEG 0911, MSMEG 5242, MSMEG 6935.*


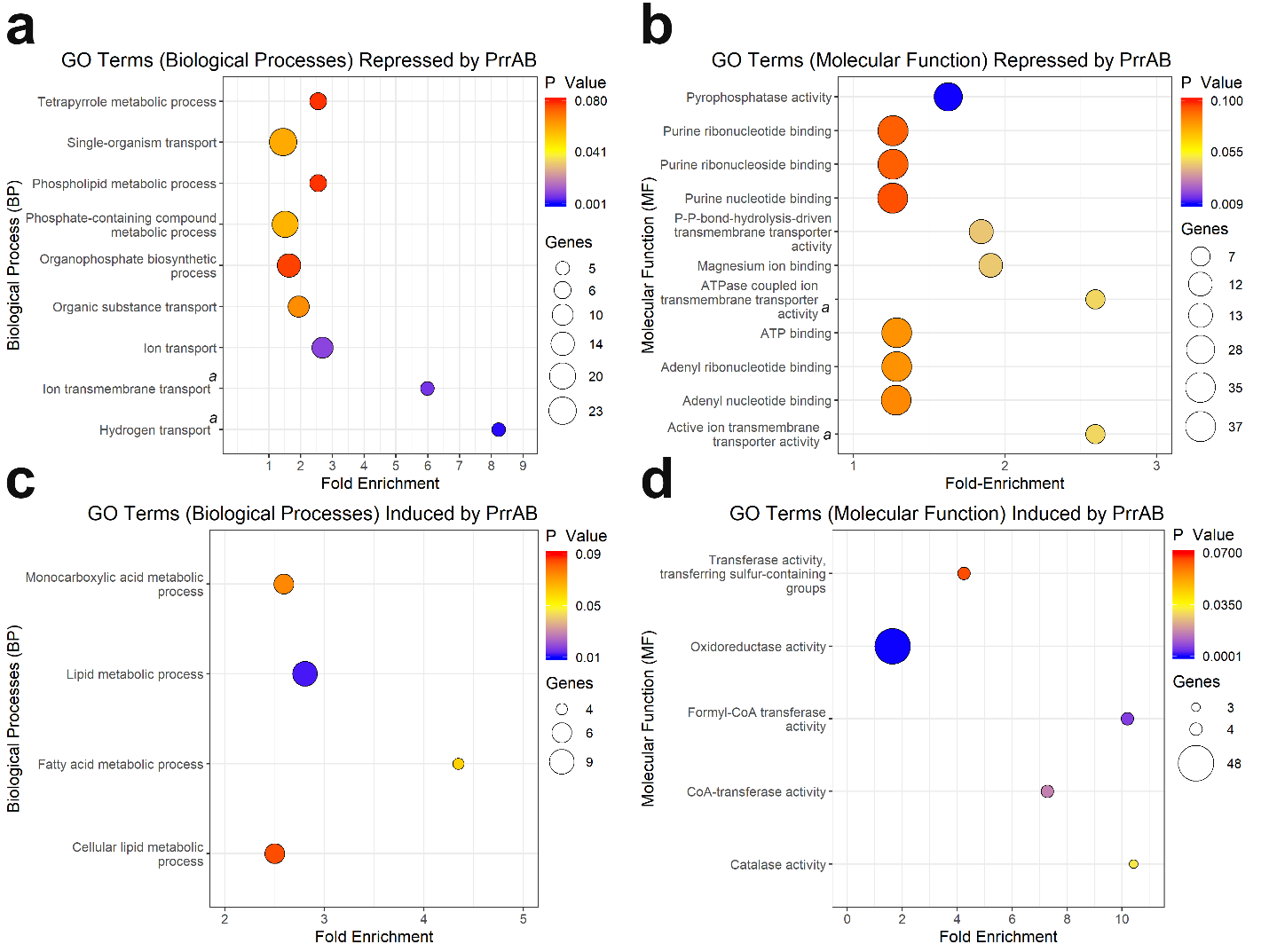


**Figure S8.** GO term enrichment associated with DEGs (*p* < 0.05) that are (**a, b**) repressed or (**c, d**) induced by PrrAB in the *prrAB* complementation background (FDL15). GO terms are categorized by (**a, c**) biological processes (BP) or (**b, d**) molecular function (MF). ^a^, GO terms share a common set of genes: *atpA, atpD, atpF, atpG,* and *atpH*).


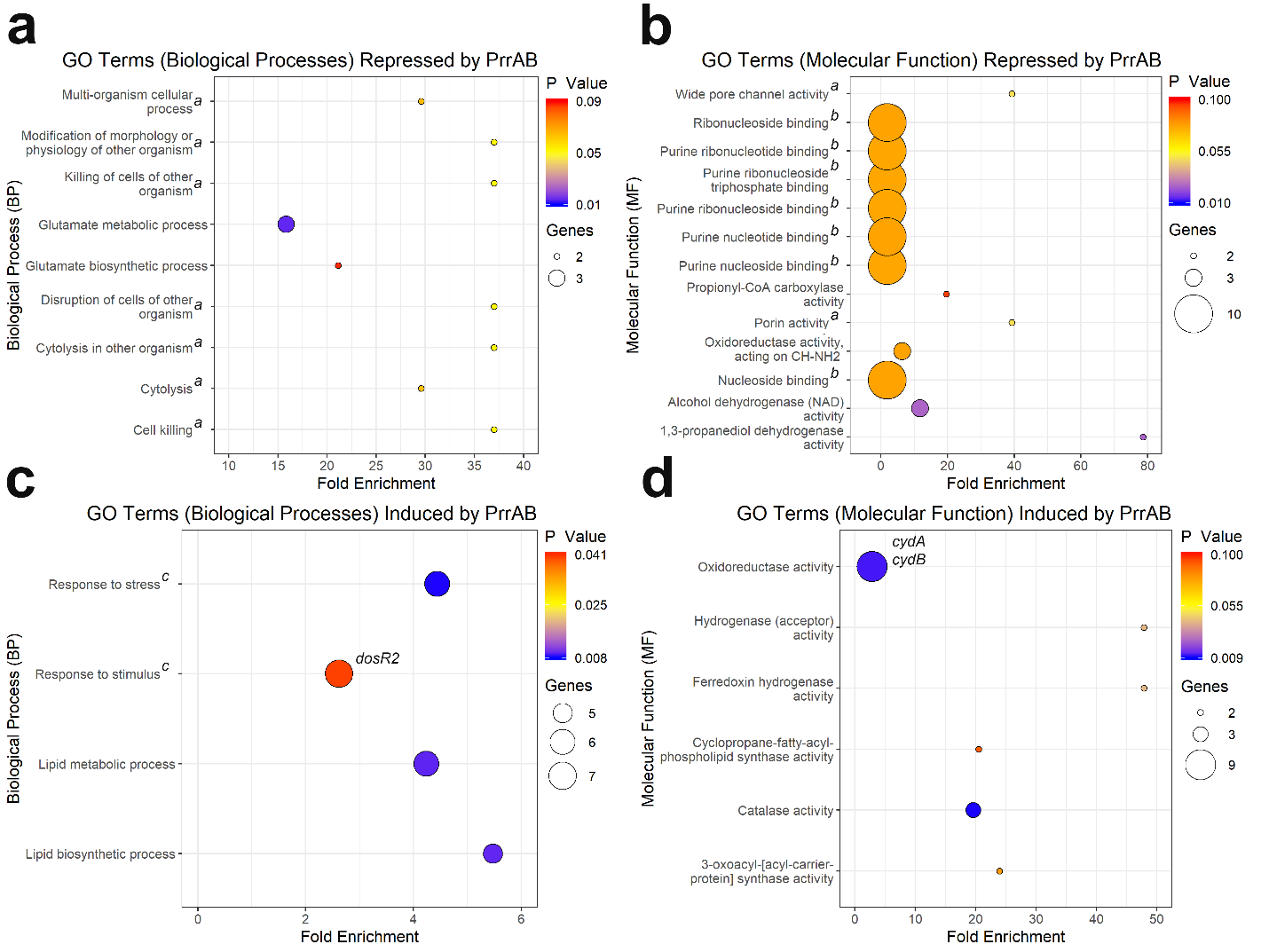


**Figure S9.** GO term enrichment associated with DEGs (*q* < 0.05) that are (**a, b**) repressed (**c, d**) or induced by PrrAB in the WT background. GO terms categorized by (**a, c**) biological processes (BP) or (**b, d**) molecular function (MF). ^a^, GO terms share a common set of genes: *MSMEG 0965* and *MSMEG 5483*. ^b^, GO terms share a common set of genes: *MSMEG 0529, MSMEG 0880, MSMEG 1807, MSMEG 1873, MSMEG 3623, MSMEG 5058, MSMEG 5659, MSMEG 5661, MSMEG 6241,* and *MSMEG 6759*. ^c^, GO terms share a common set of genes: *MSMEG 3939, MSMEG 3940, MSMEG 3945, MSMEG 5580, MSMEG 6467,* and *MSMEG 6933*.


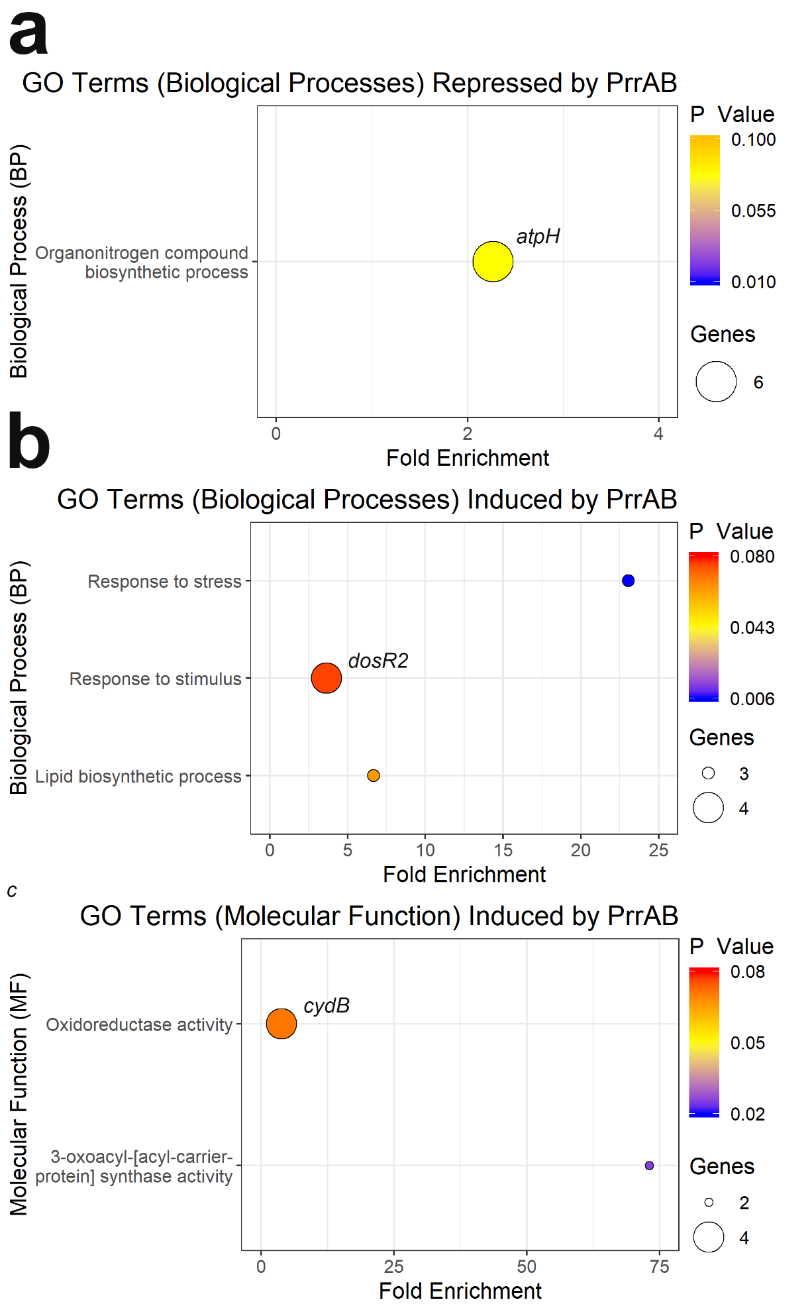


**Figure S10.** GO term enrichment associated with DEGs (*q* < 0.05) that are (**a**) repressed or (**b, c**) induced by PrrAB in the complementation background. GO terms categorized by (**a, b**) biological processes (BP) or (**c**) molecular function (MF). Note that no GO terms were returned for molecular functions in the DEG set repressed by PrrAB in the Δ*prrAB* mutant vs. complementation group comparison.


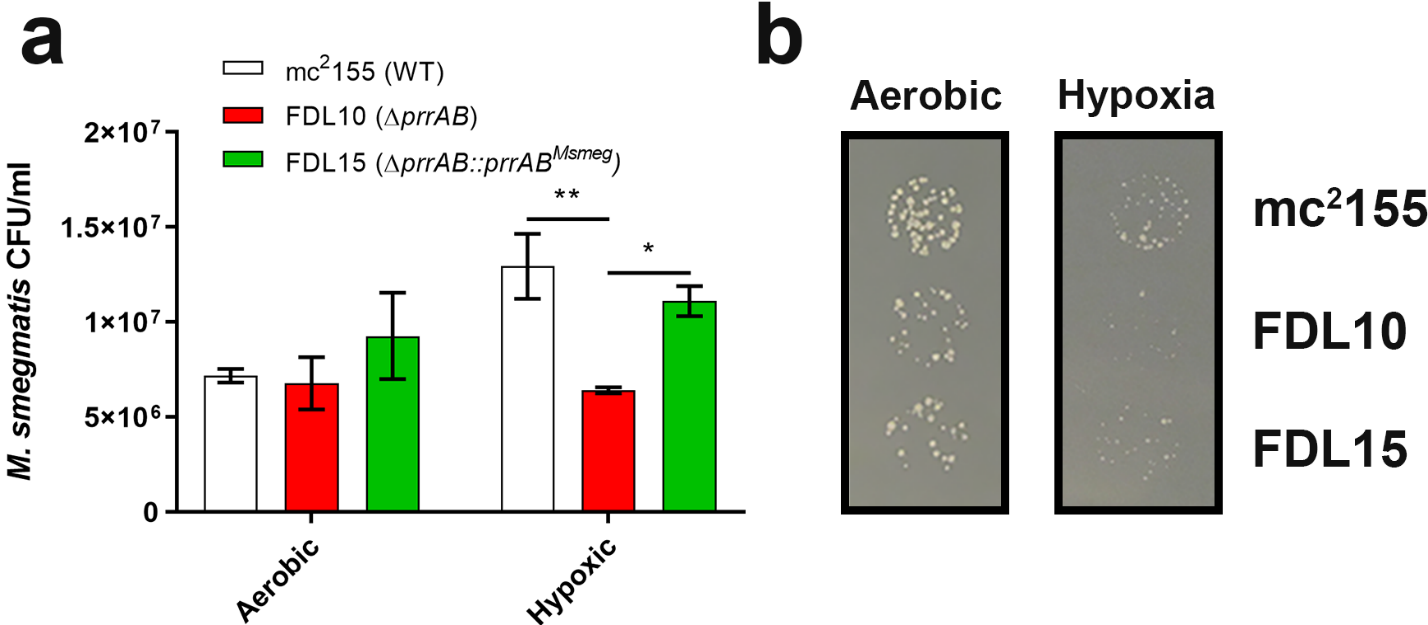


**Figure S11.** PrrAB is required for efficient growth under hypoxic conditions. (**a**) Viability of *M. smegmatis* strains after 24 h incubation in hypoxic or aerobic environments at 37ºC. (**b**) Strains were grown to mid-exponential phase (OD_600_ ~0.6), diluted, and plated onto M7H10 agar prior to incubation under aerobic or hypoxic conditions for 24 h. Images were obtained after the plates were incubated aerobically at 37ºC for an additional 48 h. *, *p* = 0.04; **, *p* = 0.0099; one-way ANOVA, Dunnett’s multiple comparisons.


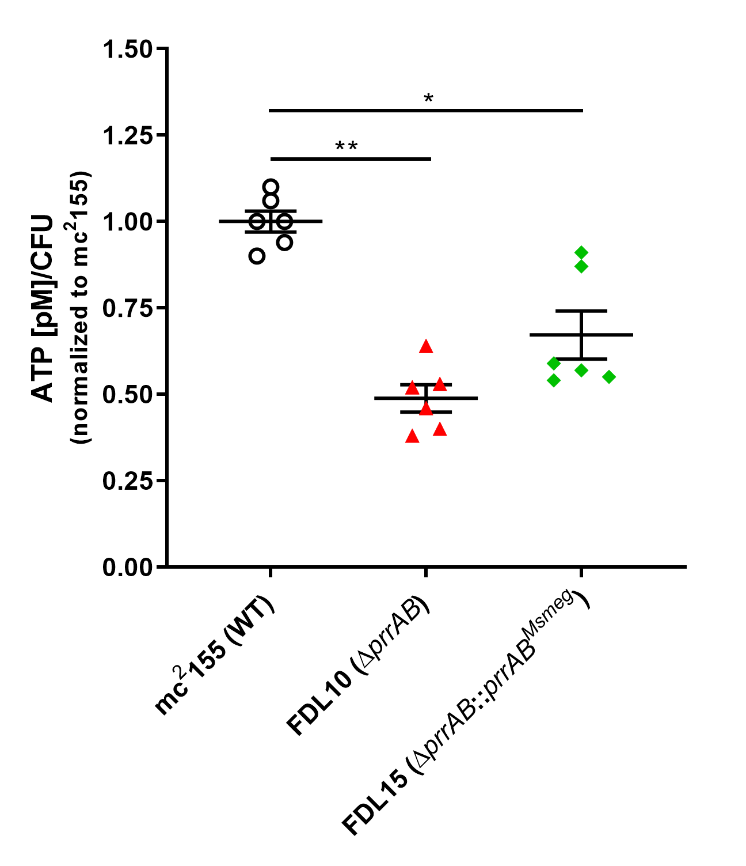


**Figure S12.** *M. smegmatis* extracellular ATP (pM/CFU) was measured from exponentially-growing (OD_600_ ~0.6) aerobic cultures in M7H9 broth. Values represent the mean ±SEM of data collected from three independent cultures. *, *p* = 0.0473; **, *p* = 0.0040; one-way ANOVA, Dunnett’s multiple comparisons.


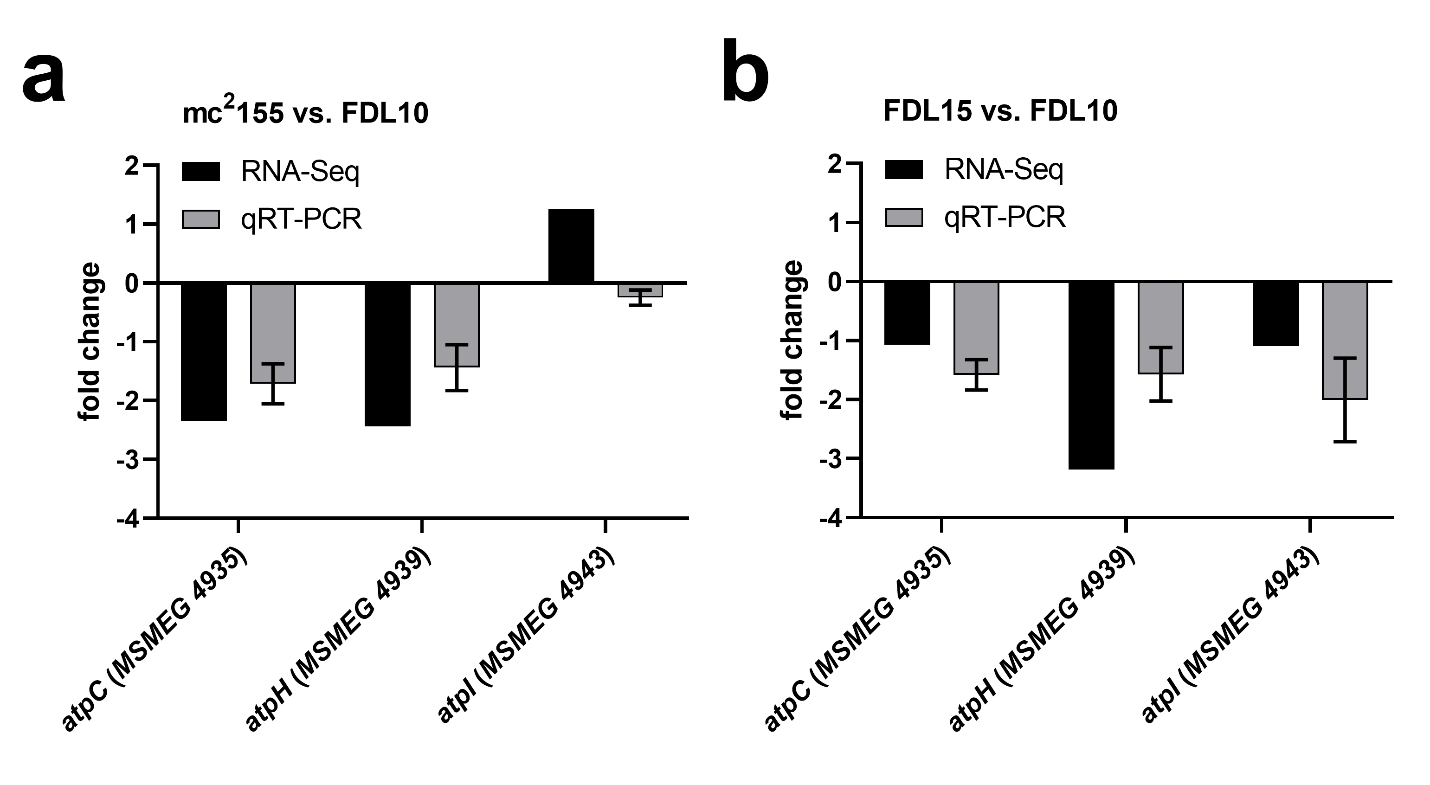


**Figure S13.** RNA-seq expression levels and qRT-PCR analysis of *atpC, atpH*, and *atpI* for (**a**) mc^2^155 vs. FDL10 and (**b**) FDL15 vs. FDL10 group comparisons. All qRT-PCR measurements were performed in triplicate using from the same RNA samples used for RNA-seq analyses. Values represent the mean ±SEM of data collected from three biological replicates. Absolute fold change ratios for qRT-PCR data were calculated using the 2­^-ΔΔCt^ method [1]. Values represent absolute fold change for both qRT-PCR and RNA-seq data.


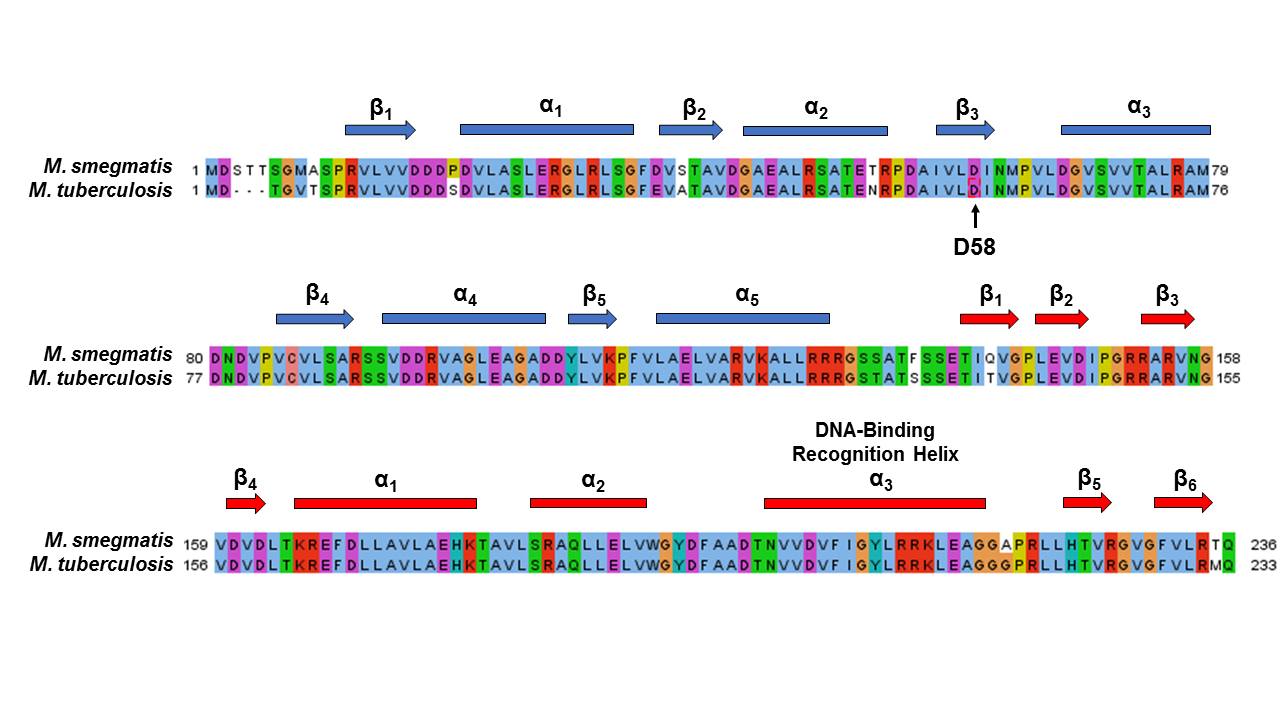


**Figure S14.** Multiple sequence alignment comparing the *M. smegmatis* and *M. tuberculosis* PrrA amino acid sequences. Secondary structures are represented by arrows (β-sheets) or bars (α-helices). Secondary structures colored in blue correspond to the N-terminal receiver domain while red corresponds to the C-terminal effector domain. The conserved phosho-receiving aspartate (D58) and DNA-binding recognition helix are labeled. Multiple sequence alignments were performed in JalView using default MUSCLE algorithms [2]. Secondary structure and DNA-binding recognition helix designations were adapted from Nowak et al. [3].

**REFERENCES**

1. Livak KJ, Schmittgen TD. Analysis of relative gene expression data using real-time quantitative PCR and the 2^-ΔΔCt^ Method. Methods. 2001. 25(4):402-408.

2. Edgar RC. MUSCLE: a multiple sequence alignment method with reduced time and space complexity. BMC Bioinformatics. 2004. 5:113. doi:10.1186/1471-2105-5-113.

3. Nowak E, Panjikar S, Konarev P, Svergun DI, Tucker PA. The structural basis of signal transduction for the response regulator PrrA from *Mycobacterium tuberculosis*. J Biol Chem. 2006. 281(14):9659-9666.
