# Supplementary material for: Comparative transcriptomics reveals PrrAB-mediated control of metabolic, respiration, energy-generating, and dormancy pathways in Mycobacterium smegmatis
Source: BMC Genomics. 2019 Dec 7;20:942. doi: 10.1186/s12864-019-6105-3 (PMC6898941; doi:10.1186/s12864-019-6105-3)
Supplement: Supplementary file 5 — Additional file 5. RNA Bioanalyzer results (RIN numbers and electrophoretic traces). [file 12864_2019_6105_MOESM5_ESM.docx]

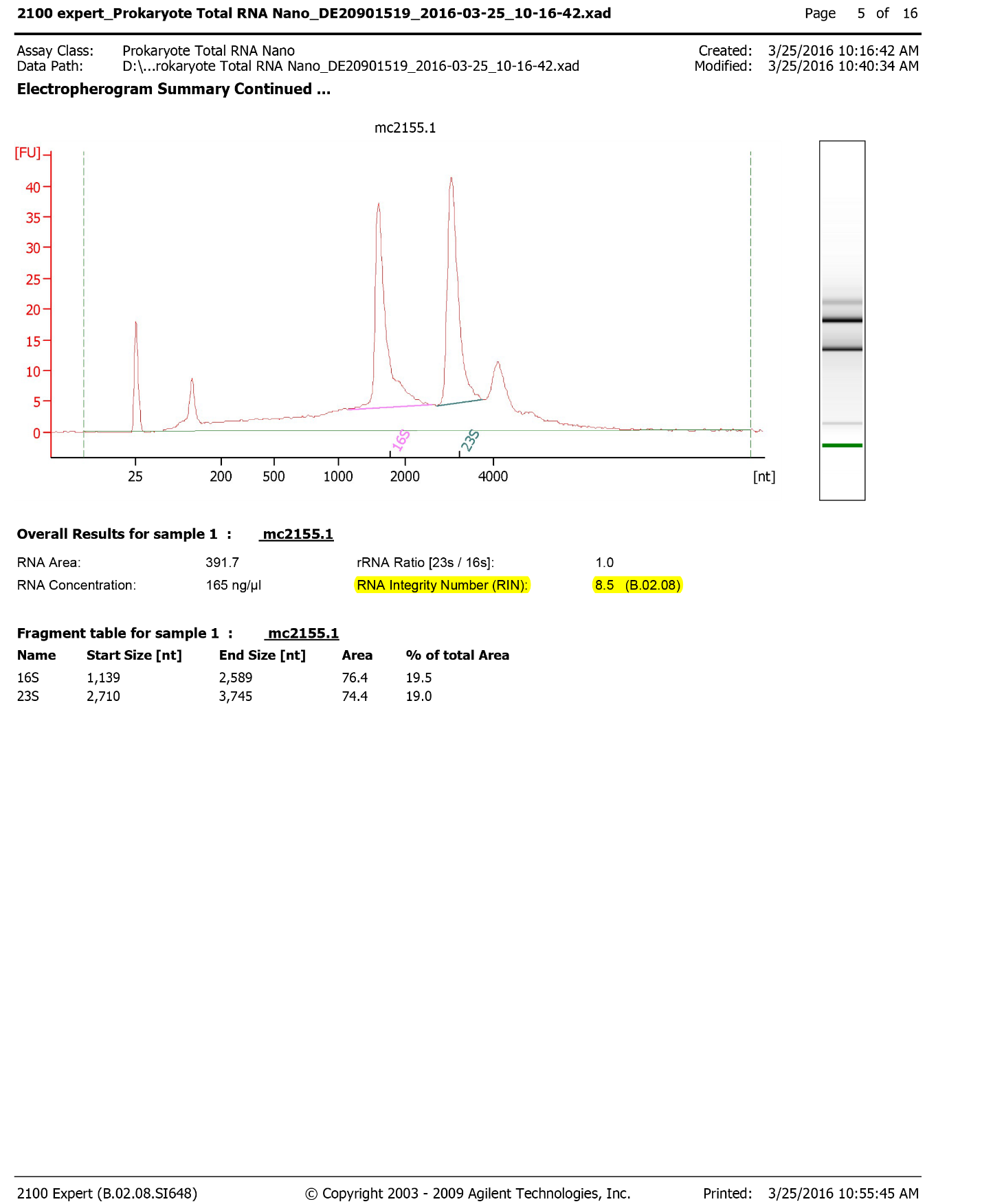


**Figure S1**. Bioanalyzer results of RNA isolated from mc^2^155 (WT), biological replicate 1 and used for next-generation sequencing. RNA integrity number (RIN) = 8.5.


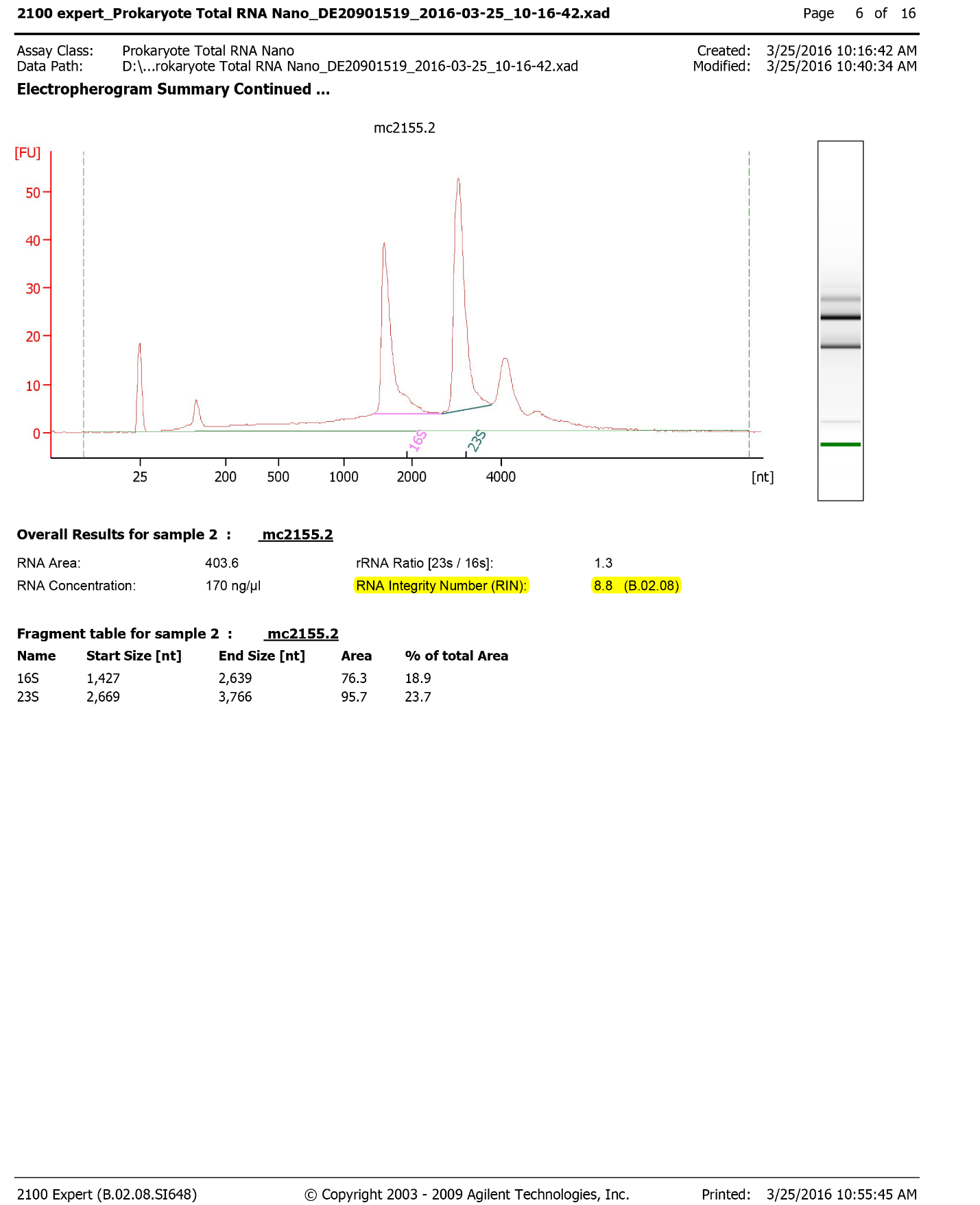


**Figure S2**. Bioanalyzer results of RNA isolated from mc^2^155 (WT), biological replicate 2 and used for next-generation sequencing. RNA integrity number (RIN) = 8.8.


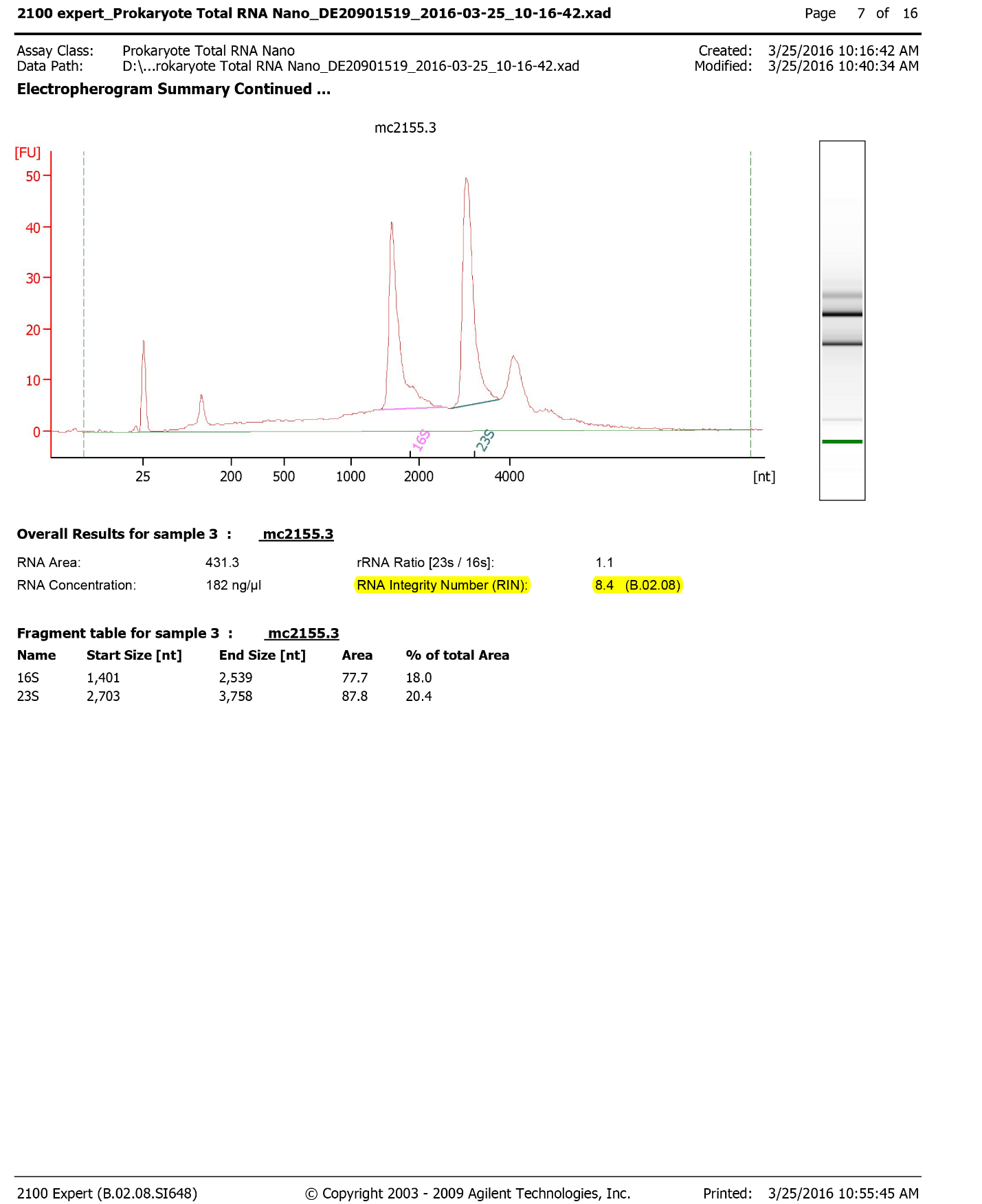
.

**Figure S3**. Bioanalyzer results of RNA isolated from mc^2^155 (WT), biological replicate 3 and used for next-generation sequencing. RNA integrity number (RIN) = 8.4.


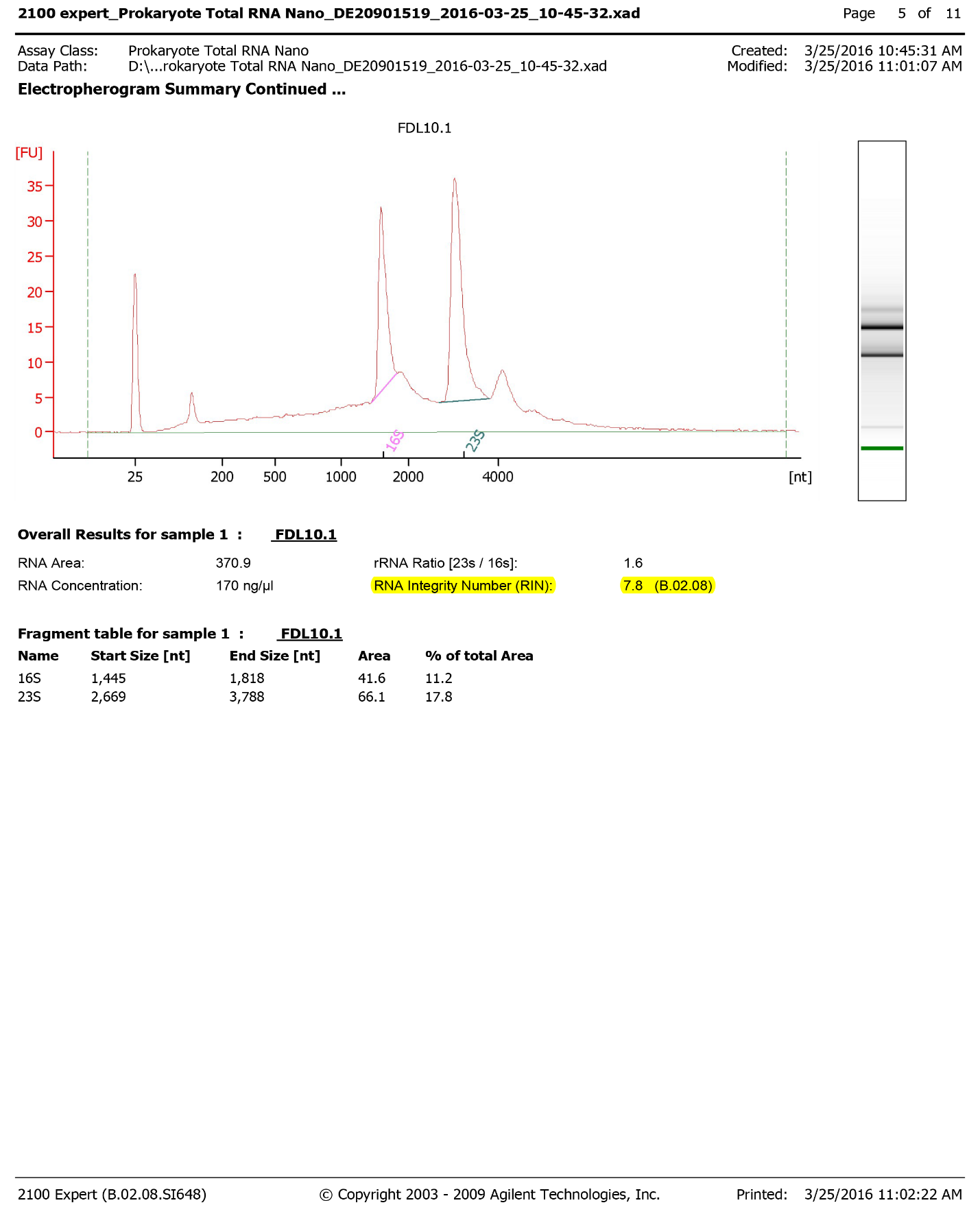


**Figure S4**. Bioanalyzer results of RNA isolated from FDL10 (Δ*prrAB* mutant), biological replicate 1 and used for next-generation sequencing. RNA integrity number (RIN) = 7.8.


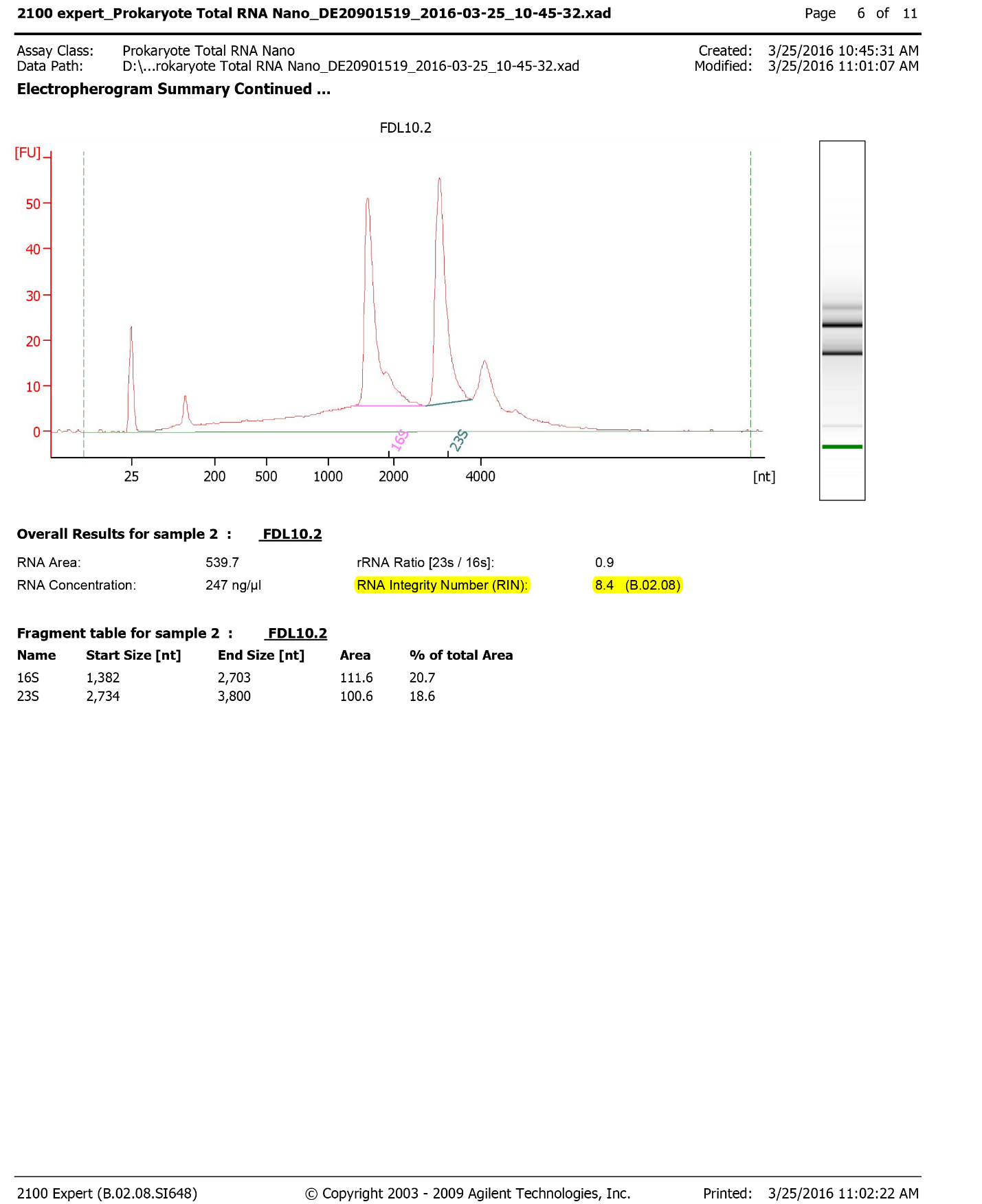


**Figure S5**. Bioanalyzer results of RNA isolated from FDL10 (Δ*prrAB* mutant), biological replicate 2 and used for next-generation sequencing. RNA integrity number (RIN) = 8.4.


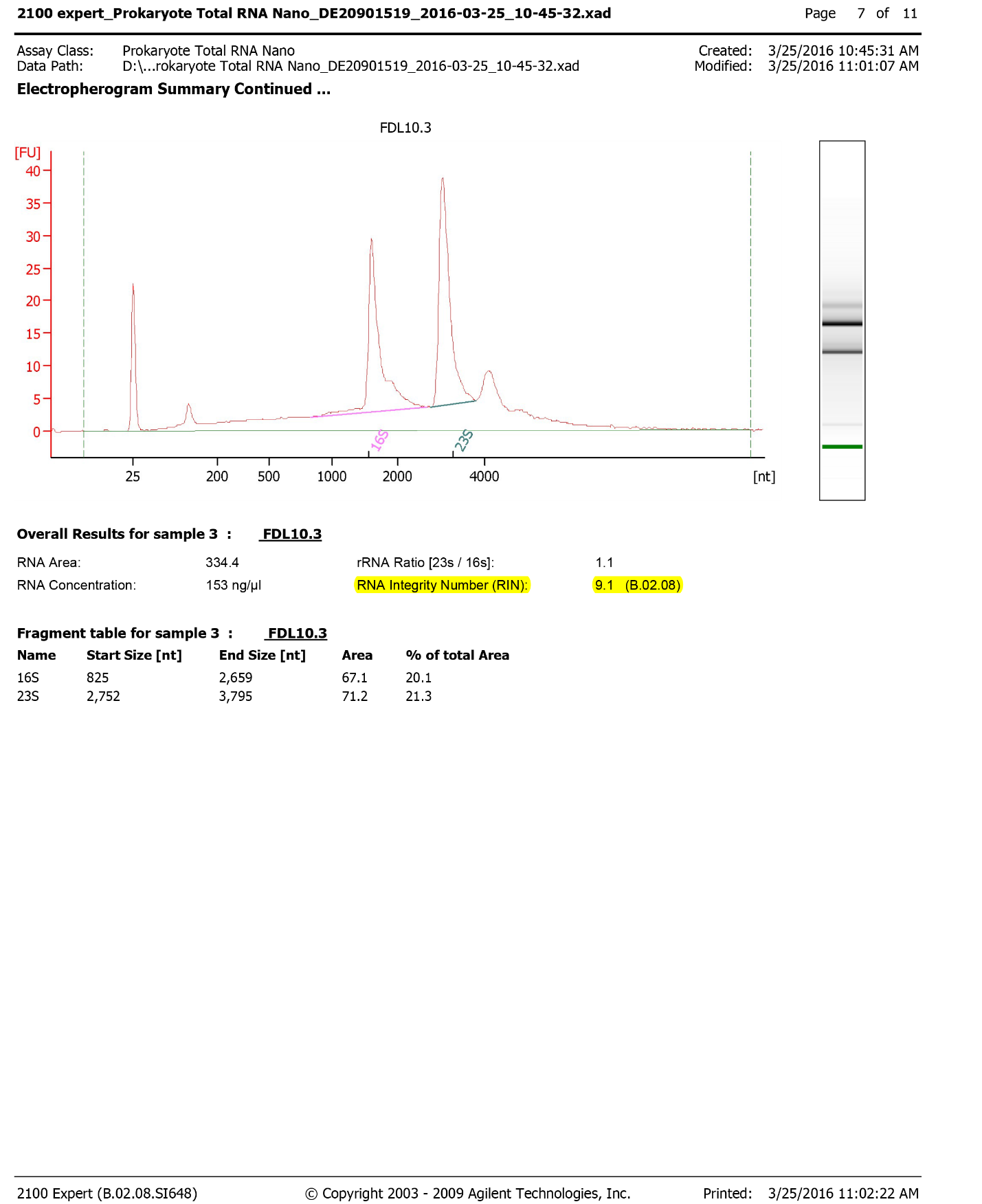


**Figure S6**. Bioanalyzer results of RNA isolated from FDL10 (Δ*prrAB* mutant), biological replicate 3 and used for next-generation sequencing. RNA integrity number (RIN) = 9.1.


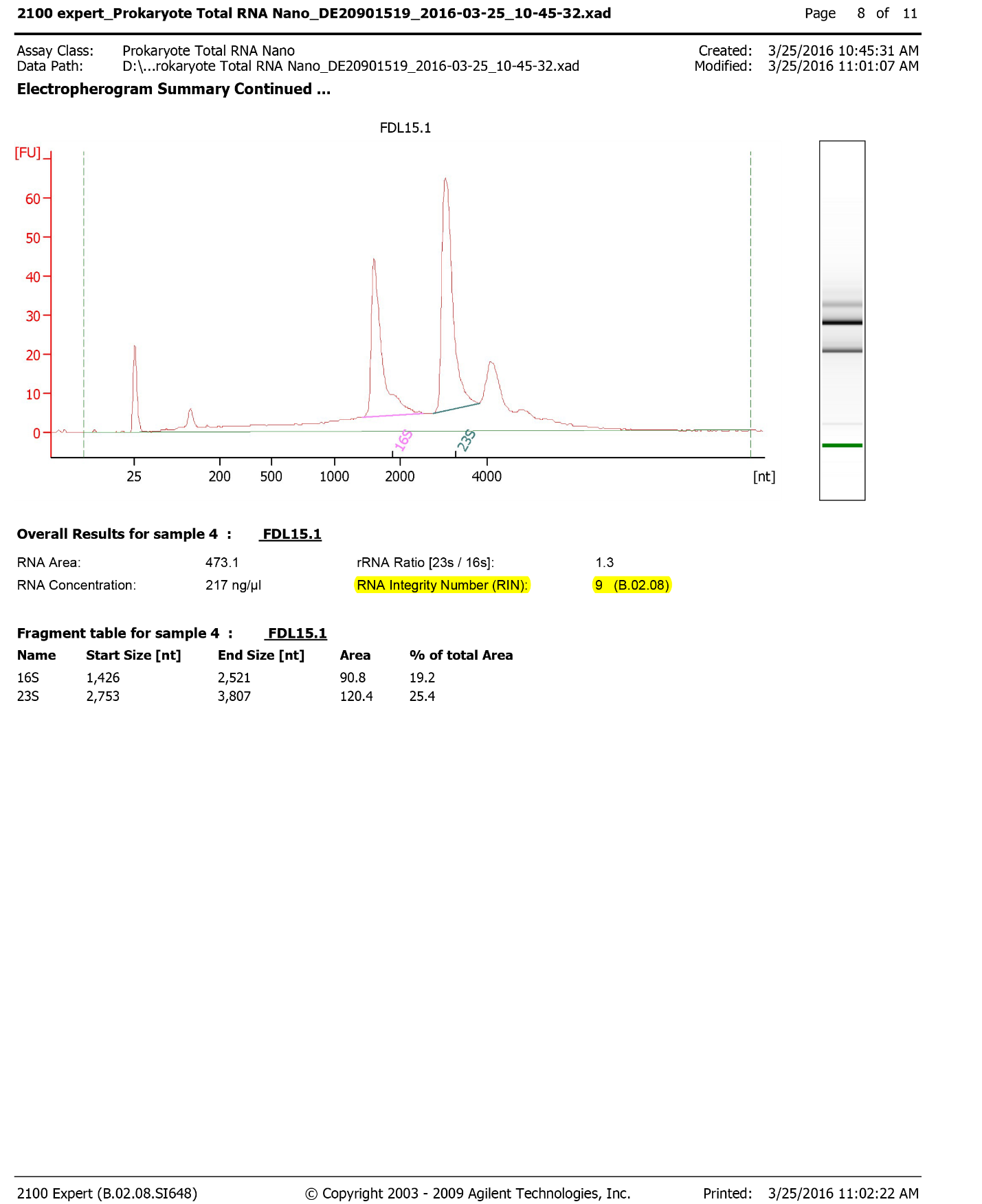


**Figure S7**. Bioanalyzer results of RNA isolated from FDL15 (complementation strain), biological replicate 1 and used for next-generation sequencing. RNA integrity number (RIN) = 9.


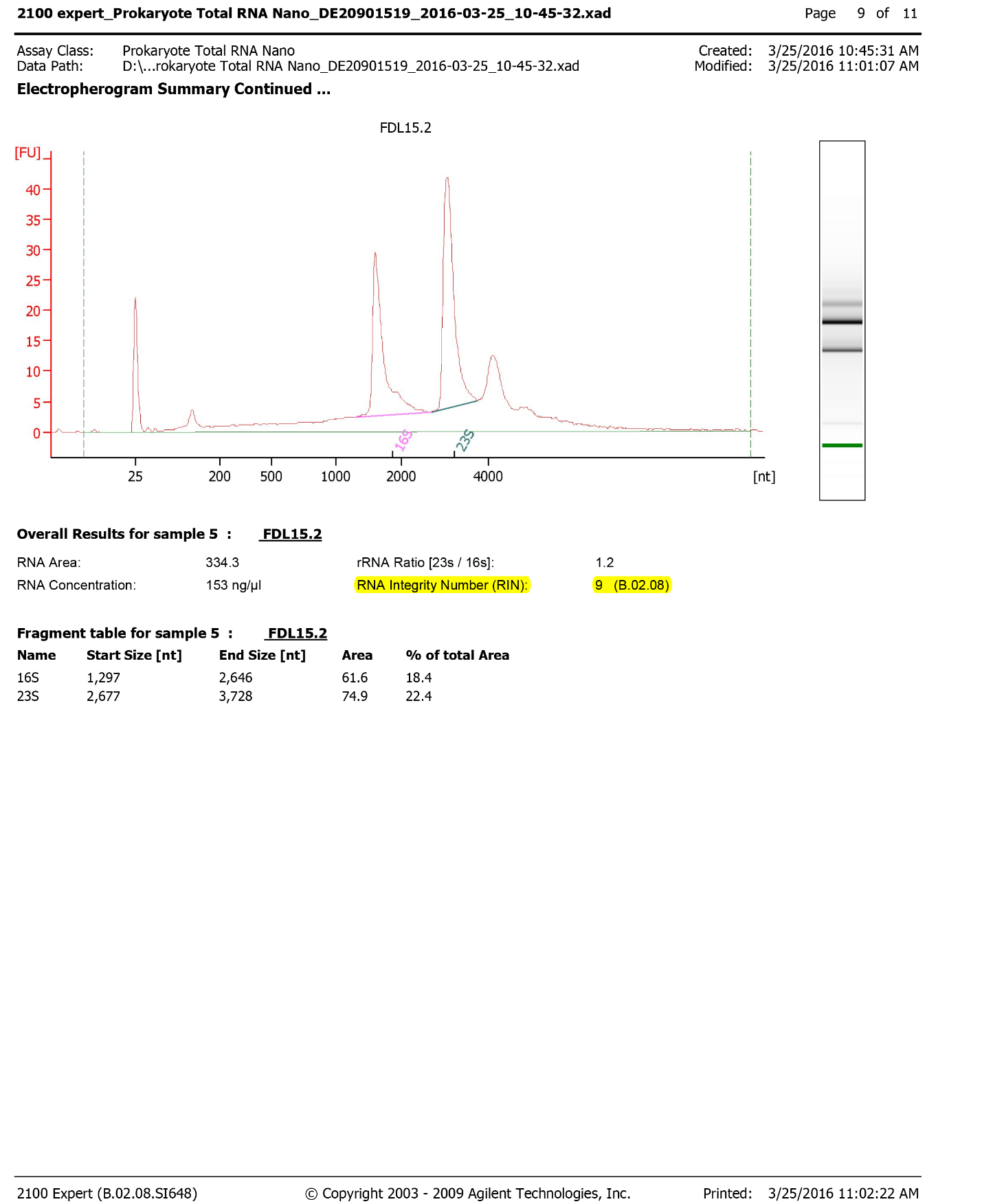


**Figure S8**. Bioanalyzer results of RNA isolated from FDL15 (complementation strain), biological replicate 2 and used for next-generation sequencing. RNA integrity number (RIN) = 9.


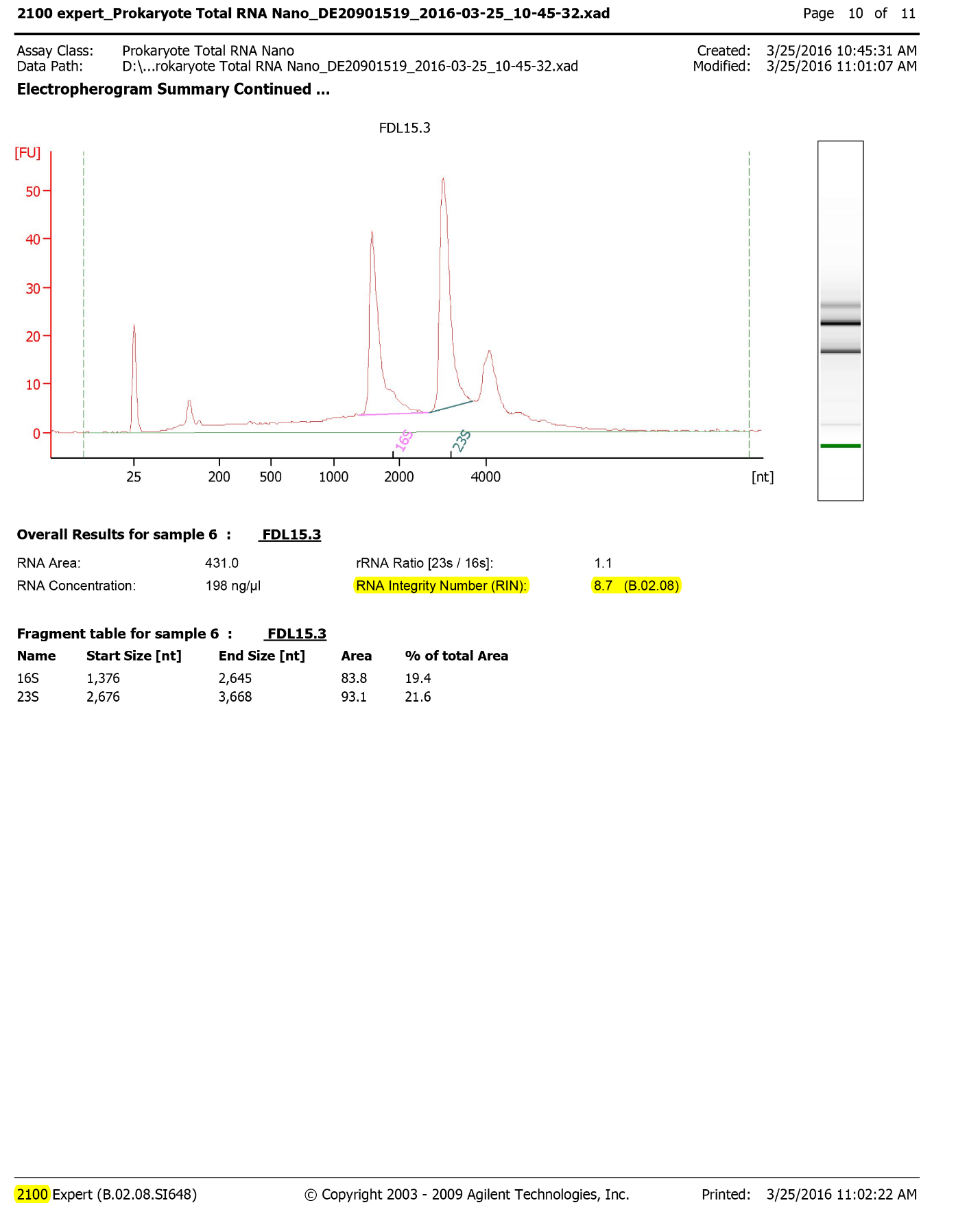


**Figure S9**. Bioanalyzer results of RNA isolated from FDL15 (complementation strain), biological replicate 3 and used for next-generation sequencing. RNA integrity number (RIN) = 8.7.
